# Spatiotemporal expression of endospore appendages and cryo-EM insights into Ena1C-mediated S-ENA anchoring in *Bacillus paranthracis*

**DOI:** 10.1038/s41598-026-38321-0

**Published:** 2026-02-03

**Authors:** Ephrem Debebe Zegeye, Mike Sleutel, Unni Lise Jonsmoen, Jingqi Chen, Luiza P. Morawska, Yohannes Beyene Mekonnen, Oscar P. Kuipers, Han Remaut, Marina Aspholm

**Affiliations:** 1 Department of Paraclinical Sciences, Faculty of Veterinary Medicine , Norwegian University of Life Sciences (NMBU) , Ås, 1433 Norway; 2 Structural Biology Brussels, Vrije Universiteit Brussel, Brussels, Belgium; 3 Structural and Molecular Microbiology Structural Biology Research Center, VIB, Brussels, Belgium; 4 Molecular Genetics Group Groningen Biomolecular Sciences and Biotechnology Institute , University of Groningen , Groningen, The Netherlands; 5 Present Address: Department of Chemistry and the Carl R. Woese Institute for Genomic Biology , University of Illinois at Urbana-Champaign , Urbana, USA

**Keywords:** *Bacillus*, Endospore appendages (ENAs), Spatiotemporal expression, Time-lapse fluorescence microscopy, Ena1C, S-ENA anchor, Biotechnology, Microbiology, Molecular biology

## Abstract

The endospores of many *Bacillus cereus* group species are decorated with highly resilient fibrous structures known as endospore appendages (ENAs), whose precise biological functions remain poorly understood. Structural and genetic studies have identified *ena1A*, *ena1B*, and *ena1C* as essential for forming the longer, thicker, and most abundant staggered (S)-ENA fibers in *Bacillus paranthracis*, whereas *ena3A* encodes the major subunit of the shorter, thinner, ladder-like (L)-ENAs. Here, we investigated the spatiotemporal expression dynamics of S- and L-ENA proteins and the specific role of Ena1C in S-ENA biogenesis. Using time-lapse fluorescence microscopy, we observed strict temporal regulation of *ena* gene expression, with no detectable ENA subunit production before spores became phase-bright. ENAs expression peaked during late sporulation phase, with fluorescence localized around the developing spore until its release; notably, S-ENA subunit expression began approximately one hour earlier than that of L-ENA subunits. Combining cryo-EM, negative-stain transmission electron microscopy, and genetic analyses, we show that Ena1C forms a nonameric ring-like structure required for tethering S-ENA to the spore surface. These findings provide new insights into the regulation of ENAs’ expression during fiber biogenesis and highlight their temporal coordination with spore coat and exosporium development.

**Supplementary Information:**

The online version contains supplementary material available at 10.1038/s41598-026-38321-0.

## Introduction

The endospores (spores) of various species within the *Bacillus cereus* group are decorated with multiple appendages (ENAs) resembling pili^1–3^. These appendages exhibit remarkable mechanical robustness^4^ and resistance to chemical and enzymatic treatments^5,6^. Previous efforts to characterize the protein composition of ENAs via mass spectrometry were unsuccessful, owing to their exceptional resistance to conventional solubilization and enzymatic degradation methods. However, recent cryo-electron transmission microscopy (Cryo-EM) advancements have enabled the determination of the three-dimensional architecture of three different types of ENAs^5–7^. These structural discoveries, in turn, enabled the structure-guided identification of the genes encoding the ENA proteins^5,6^.

Although appendages on spore surfaces were first observed nearly 50 years ago^1,8^, the precise biological functions of ENAs have remained elusive. Previous studies hypothesized that ENAs may play roles in spore adhesion to biotic and abiotic surfaces, spore aggregation, and biofilm formation (reviewed in)^9^, but direct functional evidence was lacking. Recent investigations have shown that ENAs contribute to spore-spore aggregation and surface adhesion, providing new insights into their role in spore biology^4,10^. One study further demonstrated that wildtype *B. paranthracis* spores expressing ENAs attach significantly stronger to industrially relevant surfaces, such as stainless steel and polypropylene, than ENA-deficient mutant spores, suggesting that ENAs contribute directly to the persistence of spores in industrial environments^11^.

Based on the arrangement of the subunits within the fibers, two distinct ENA morphologies, known as Staggered (S)-ENA and Ladder (L)-ENA, have been characterized in the *B. paranthracis* food poisoning outbreak strain NVH 0075/95 (hereafter referred to as *B. paranthracis*) (Supplementary Fig. S1)^5^. S-ENA fibers constitute approximately 90% of the appendages on the surface of spores of this strain and are encoded by the bicistronic operon *ena1AB*^5^. However, the genes that encode the 4–6 tip fibrillae (also known as ruffles) remain unidentified. The assembly of S-ENA fibers involves the polymerization of Ena1A and Ena1B subunits, forming a structure that extends from the spore coat and traverses the exosporial sac^5^. The *ena1C* gene, which is located adjacent to *ena1B* in the opposite orientation (Supplementary Fig. S1a), is essential for the formation of S-ENA, as its deletion results in spores lacking S-ENA^5^. However, Ena1C does not appear to contribute to the stalk structure itself, leading to the hypothesis that Ena1C may instead function in assembly and/or anchoring of S-ENA to the spore body.

The L-ENA fiber accounts for approximately 10% of the total number of ENA fibers present on the surface of *B. paranthracis* spores. It is encoded by the bicistronic *bclA-ena3A* operon, located either on the chromosome, on a plasmid, or in some cases on both, where it appears as paralogs^6^. Whereas the *ena3A* gene constitutes the major subunit of the L-ENA fiber stalk, the tip fibrillum (ruffle) is encoded by the *l-bclA* gene (Supplementary Fig. S1b)^6^. Furthermore, the *exsL* gene, located upstream of *l-bclA*, has been identified as a crucial tethering component for the L-ENA fiber; its deletion results in loss of L-ENA fibers attached to the exosporium^6^. Quantitative real-time PCR analyses demonstrated that the expression of genes encoding both S-ENA and L-ENA fibers increases during the sporulation phase^5,6^. However, it remains unclear at what stage of sporulation ENA proteins are expressed, whether S- and L-ENA subunit proteins are expressed simultaneously or sequentially, and where within the sporulating cell this expression occurs. Moreover, the precise role of *ena1C* in S-ENA biogenesis is not yet understood.

Here, to gain deeper insights into the spatiotemporal expression dynamics of S-ENA and L-ENA subunits and to investigate the specific role of Ena1C in S-ENA biogenesis, we combined time-lapse microscopy of fluorescent protein–tagged Ena1A, Ena1B, Ena1C, and Ena3A with negative-stain transmission electron microscopy (nsTEM), cryo-EM, AlphaFold modeling, and targeted genetic analyses. These complementary approaches enabled precise mapping of ENA expression onsets and progression during spore development, providing a detailed temporal framework for appendage formation, and revealing structural features of Ena1C that underlie its role in S-ENA biogenesis.

## Materials and methods

### Chromosomal integration of *ena* genes with fluorescent protein-encoding genes

The fusion of *ena1A* and *ena1C* with the superfolder GFP gene (*sfGFP*) at the C-termini, as well as the tagging of *ena3A* with the mKate gene (*mKate2*), was achieved using the markerless gene replacement technique^12,13^. The fluorescent proteins used were SPS6 and KPS12 variants of sfGFP and mKate2, respectively, which were chosen for their enhanced brightness in *Bacillus* species^14^. Briefly, the constructs contain the open reading frame (ORF) of the respective *ena* gene (excluding the stop codon), seamlessly fused with a GFP linker sequence (5’-GGTAGCGGTGGAGGTGGCAGC-3’) and a fluorescent gene in tandem. This sequence was flanked by upstream and downstream homology arms specific to the *ena* gene. The lengths of the corresponding upstream and downstream homology sequences, respectively, were 704 bp and 704 bp for *ena1A*, 699 bp and 700 bp for *ena1C*, and 708 bp and 677 bp for *ena3A*. Synthetic gene constructs, flanked by EcoRI restriction sites, were ordered from Synbio Technologies (U.S.A.). The constructs were digested with EcoRI and ligated into the pMAD-I-SceI shuttle vector (Supplementary Table S1). The resulting pMAD constructs (Supplementary Table S1) were transformed into *B. paranthracis*^5^, and blue colonies were selected. The selected colonies were further transformed with pBKJ233 to facilitate double crossover, as previously described^12,13^. White colonies were randomly picked and screened by colony PCR (primers shown in Supplementary Table S2) for expected band sizes, and positive candidates were confirmed by Sanger sequencing. The *B. paranthracis* constructs generated for this study are summarized in Table 1.

**Table 1:** List of *B. paranthracis* constructs used in this study.

Strain/genotype	Description	Source
*B. paranthracis*	Wildtype	^5^
*B. paranthracis ena1A-sfGFP*	Chromosomally integrated	This study
*B. paranthracis ena1C-sfGFP*	Chromosomally integrated	This study
*B. paranthracis ena3A-mKate2*	Chromosomally integrated	This study
*B. paranthracis*::pHT304*-P*_*ena1AB*_*-sfGFP-ena1B*	Plasmid expression	This study
*B. paranthracis*::pHT304*-P*_*ena1AB*_*-ena1B-sfGFP*	Plasmid expression	This study
*B. paranthracis*::pHT304*-P*_*ena3A*_*-mKate2-ena3A*	Plasmid expression	This study
*B. paranthracis*::pHT304*-P*_*ena3A*_*-mKate2-ena1C*	Plasmid expression	This study
*B. paranthracis ena1A-sfGFP*::pHT304*-P*_*ena3A*_*-mKate2-ena3A*	Co-expression of Ena1A and Ena3A	This study
*B. paranthracis ena1C-sfGFP*::pHT304*-P*_*ena3A*_*-mKate2-ena3A*	Co-expression of Ena1C and Ena3A	This study
*B. paranthracis*::pHT304	Empty vector (control)	This study
*B. paranthracis* Δ*ena1C*	*ena1C* knockout strain	^5^
*B. paranthracis*::pHT304*-P*_*ena1C*_*-ena1C*	Ena1C overexpressing strain	This study
*B. paranthracis* Δ*ena1C*:: pHT304*-P*_*ena1C*_*-ena1C* (C142S)	*ena1C* knockout strain complemented with *ena1C* (C142S)	This study

### Expression of fluorescently tagged ENA subunits from low-copy plasmid

The low-copy number shuttle vector pHT304^15^ was used to express the *ena* genes fused with fluorescent protein tags, *sfGFP* or *mKate2* (Supplementary Table S1). Unless indicated otherwise, *sfGFP* (SPS6) and *mKate2* (KPS12)^14^ genes were fused in-frame to the 5ꞌ end of the respective *ena* coding sequence, yielding N-terminally tagged constructs expressed from their native promoters (Supplementary Fig. S2). The DNA sequences comprising the native promoters of the respective *ena* gene, the ORF of the selected fluorescent gene, the GFP linker sequence, the ORF of the specific *ena* gene, and the stop codon in tandem, flanked by EcoRI restriction sites, was synthesized at Synbio Technologies (U.S.A.). The amino acid sequences of the fusion proteins expressed are given in Supplementary Table S3.

### Manual time-course fluorescence microscopy

A single colony of *B. paranthracis* expressing a fluorescent protein tagged ENA subunit was picked from an LB agar plate and inoculated into 10 mL of brain heart infusion (BHI) medium in a 50 mL conical tube. The cultures were incubated at 37 °C overnight with shaking at 200 rpm. The next day, 0.5 mL of the overnight culture was transferred to a 250 mL Erlenmeyer flask containing 50 mL of sporulation medium. The sporulation medium^5^ consisted of 8 g/l bacto nutrient broth (Difco), 1 µM FeSO_4_, 2.5 µM CuCl_2_, 12.5 µM ZnCl_2_, 66 µM MnSO_4_, 1 M MgCl_2_, 5 mM (NH4)_2_SO_4_, 2.5 µM Na_2_MoO_4_, 2.5 µM CoCl_2_, 1 mM Ca (NO_3_)_2_, pH 7.6. To monitor sporulation and protein expression dynamics, 1 mL of the culture was collected hourly for fluorescence microscopy analysis. Prior to microscopy analysis, a thin layer of 1.5% agarose (w/v) (Serva # 11404.05) was solidified on a 10-well multitest slide (MP Biomedicals, # 096041805E). One µL of the culture sample was transferred onto the agarose pad, briefly incubated at room temperature to immobilize the cells, covered with a standard coverslip, and examined by fluorescence microscopy as described in the section *Fluorescence microscopy* below. Where indicated, 24-h cultures were harvested by centrifugation at 4000 × g for 10 min, washed once with PBS (pH 7.2), and the final pellet was resuspended in PBS for fluorescence microscopy.

### Automated time-lapse fluorescence microscopy

To enable growth and sporulation of *B. paranthracis* for automated time-lapse microscopy, we cultivated the bacteria on agarose patches on microscope slides as previously described^16^, with modifications. Specifically, we used a modified sporulation agar in which the standard sporulation medium^5^ was adjusted by reducing the nutrient broth (Difco) concentration from 8 g/L to 2 g/L. Single colonies of each strain were then inoculated into 15 mL of the modified sporulation medium in 50 mL conical tubes and incubated at 37 °C with shaking (200 rpm) for 24 h. Following incubation, cultures were centrifuged, and supernatants sterilized by filtration through 0.2 μm membrane filters.

The modified sporulation agar was thus prepared combining 250 µL of the adjusted sporulation medium, 1 mL of 3% melted sterile agarose, and 0.75 mL of the filtered 24-h culture supernatant, yielding a total volume of 2 mL. This mixture was briefly warmed in a microwave to ensure homogeneity. For strains carrying the low-copy plasmid pHT304, erythromycin was added to a final concentration of 5 µg/mL. To assemble the slides, a Gene Frame (1.5 × 1.6 cm; Thermo Scientific #AB0577) was attached to a sterile microscope slide, and approximately 100 µL of the melted agarose medium was pipetted into the well. A second sterile slide was immediately placed on top, and the assembly was allowed to solidify at 4 °C (Supplementary Fig. S3). Prior to inoculation, the slides were pre-incubated at 37 °C for 20 min to stabilize the conditions.

To facilitate aeration, the top slide was carefully removed, and a third of the central segment of the agar was excised using a sterile scalpel blade (Merck #S2646). For inoculation, single colonies of each strain were grown overnight in BHI broth and 60 µL of the culture was transferred to fresh modified sporulation medium and incubated for 1 h for adaptation. Subsequently, 1.5 µL of the culture (OD_600_ ≈0.01) was spotted onto three distinct locations on the agar surface. A sterile coverslip was applied to seal the preparation, ensuring firm contact at the edges (Supplementary Fig. S3). The inoculated slides were immediately transferred to the time-lapse fluorescence microscopy setup, with imaging parameters detailed in the *Fluorescence Microscopy* section.

### Fluorescence microscopy

Deltavision elite deconvolution microscope (Applied Precision, GE Healthcare, Issaquah, WA, USA), equipped with an environmental chamber, was used for time-lapse imaging. Images were captured using a 100x phase-contrast objective (oil immersion, Deltavision; Olympus, Japan). Standard fluorescence filter sets were used to visualize sfGFP and mKate2. Unless otherwise stated, the imaging settings were as follows: POL (exposure 0.5 s, and 32% transmission), GFP (exposure 0.3 s, 10% transmission), and mCherry (exposure 1 s, 10% transmission). For *B. paranthracis ena1C*-sfGFP constructs, the exposure time was increased to 1 s. In automated time-lapse imaging, 4 or 5 isolated cells were marked for tracking, and imaging was conducted with 10–12 min intervals at 37 °C until the sporulation process was completed (~ 20–24 h). For 24-h flask culture experiments, images were acquired using Nikon ECLIPSE Ti2 inverted microscope equipped with a 100x oil-immersion objective (Plan Apo λ 100×/1.45 oil, Nikon). Phase contrast and fluorescence images were captured using a Nikon DS-Qi2 monochrome camera. Image acquisition and exposure control were performed using NIS-Elements AR software (Nikon). The exposure times for each imaging modality were as follows: phase-contrast, 1 s; FITC fluorescence, 1 s; and mCherry fluorescence, 1s.

### Negative-stain transmission electron microscopy (nsTEM)

To prepare samples for nsTEM imaging, spores were scraped off 3 to 4-week-old blood agar plates and suspended in distilled water. The spores were washed three times by repeated resuspending and centrifugation. Where indicated, spores were instead directly resuspended in water and examined for the presence of unattached S-ENAs in the environment. A 400-mesh copper grid covered with FCF400-CU Formvar Carbon Film was placed onto a droplet of the suspended spores for 1 min. The excess suspension was removed by capillary action using a dry filter paper. The grid was then placed on a droplet of 4% uranyl acetate for negative straining for 1 min. After removing the excess stain with filter paper and allowing the grid to air-dry, the samples were analyzed using a JEM-2100Plus Electron Microscope (JEOL Ltd.).

### Image analysis

Fluorescence intensity analysis, color assignment, overlay images, and time-lapse videos were created using Fiji software (https://imagej.net/Fiji). Corrected total spore fluorescence was analyzed as described previously^17^. The length and number of ENAs were determined by analyzing nsTEM images using the measuring tool in the Fiji software. The number of pixels measured were converted to nm by using the number of pixels/nm from the scalebar as a conversion factor.

### Recombinant expression of His_6_-tagged Ena1C

A synthetic open reading frame encoding Ena1C and Ena1C (∆1–36) with an N-terminal 6xHis-tag followed by a TEV cleavage site (ENLYFQG) were codon-optimized for recombinant expression in *E. coli*, synthesized by Twist Biosciences, and cloned into the pET28a expression vector (see Supplementary Table S3 for the amino acid sequences). Large-scale recombinant expression of Ena1C was carried out in the T7 Express lysY/Iq *E. coli* strain from NEB. A single colony was inoculated into 200 mL of LB and grown at 37 °C with shaking at 150 rpm overnight to obtain the primary culture. Next morning, 6 L of LB was inoculated with 20 mL/L of primary culture and grown at 37 °C with shaking until the OD_600_ reached 0.8, after which protein expression was induced with 1 mM isopropyl β‐D‐1‐thiogalactopyranoside (IPTG). The culture was incubated for a further 3 h at 37 °C and harvested by centrifugation at 5,000 rcf. The whole‐cell pellet was resuspended in lysis buffer (20 mM potassium phosphate, 500 mM NaCl, 10 mM β‐mercaptoethanol, 20 mM imidazole, 8 M urea, pH 7.5). The lysate was centrifuged to pellet sacculi, and membrane envelopes at 40,000 rcf for 45 min. The cleared lysate was passed over a 5 mL HisTrap HP column (GE Healthcare) and eluted with 20 mM potassium phosphate, pH 7.5, 8 M urea, 250 mM imidazole in gradient mode (20–250 mM imidazole) (Supplementary Fig. S15a). Fractions were further analyzed using SDS–PAGE to check for purity (Supplementary Fig. S15b). Selected fractions were pooled, concentrated to 500 µL, dialyzed against 25 mM potassium phosphate pH 7.5, 50 mM NaCl and passed over a BioRad ENrich™ Sects. 650 10 × 300 column for removal of aggregated species. Selected fractions were pooled and concentrated to 1 mg/mL.

### Cryo-electron transmission microscopy

QUANTIFOIL^®^ holey Cu 400 mesh grids with 2-µm holes and 1‐µm spacing were glow discharged in vacuum using plasma current of 5 mA for 1 min (ELMO; Agar Scientific). Right before cryo‐plunging, 3 µL of a 1 mg/mL Ena1C sample was applied on the holey grid at 95% humidity and room temperature in a Gatan CP3 cryo‐plunger. The grid was machine‐blotted with Whatman grade 2 filter paper for 3.5 s from both sides and plunge frozen into liquid ethane at −176 °C. Grids were stored in liquid nitrogen until data collection. High‐resolution cryo-EM movies at 5760 × 4092 pixels were recorded using SerialEM^18^ on a JEOL CRYO ARM 300 microscope equipped with an omega energy filter (operated at slit width of 20 eV). 9137 movies were captured with a K2 direct electron detector run in counting mode at a magnification of 60 K with a calibrated pixel size of 0.695 Å/pixel, and a total exposure of ~ 60 e^−^/Å^2^ over 60 frames. Movies were imported into cryoSPARC^19^ v4.7.1 for further processing. Movies were motion-corrected using Patch Motion Correction and defocus values were determined using Patch CTF. Exposures were curated and particles from 200 micrographs were picked using the blob picker with 75Å−120Å minimum and maximum diameter, and extracted with a box size of 52 × 52 pixels, with a pixel size of 2.78Å/pixel. The resulting particle stack was subjected to 2D classification and selected class averages were used in a subsequent Template Picker job of the entire dataset. After Micrograph Junk Detection, this yielded ~ 2.59 million particles, which after several rounds of 2D classification was filtered down to 1.15 million particles. 4 initial volumes were generated with the Ab-Initio job, 3 of which were selected as inputs in subsequent Heterogeneous Refinement. 726,988 particles corresponding to a single heterogeneous model were classified further in a 3D Classification job with 10 classes. 486,293 particles corresponding to 5 selected classes were used as input for Homogeneous refinement with global C9 imposed, re-extracted to a pixel size of 1.39Å/pixel and used in a final homogenous refinement in C9. Despite best efforts to enrich particles corresponding to tilt and side-view orientations in the final particle stack, the resulting volume suffers from clear missing wedge artefacts. As an initial atomic model, we used a nonameric AlphaFold3^20^ prediction of D1-33 Ena1C. Regions of the input model that were not supported by clear density were removed. Due to pronounced map anisotropy characterized by a cFAR value of 0.16, we limited model refinement to rigid-body fitting into the cryo-EM volume using ChimeraX^21^ followed by a single round of refinement with phenix.refine^22^ with the “rigid_body” flag enabled. Figures were created using ChimeraX using an EMReady^23^ postprocessed map which was deposited to the EMDB alongside the B-factor sharpened map (Supplementary Table S4).

### Statistical analyses

Statistical analyses were performed using GraphPad Prism version 10. Unpaired two-tailed t-tests and ordinary one-way ANOVA with multiple comparisons (comparing the mean of each group with the mean of every other group) were conducted to evaluate the statistical significance between groups. P-values < 0.05 were considered statistically significant.

## Results

### Optimization of growth and sporulation of *B. paranthracis* for time-lapse microscopy

To enable time-lapse microscopy of *B. paranthracis* sporulation, we first sought to establish a robust method for growing and sporulating the cells directly on a microscope slide (Supplementary Fig. S3). However, initial attempts to grow and sporulate *B. paranthracis* on an agarose patch placed on a microscopic slide using the standard Sporulation medium^5^ were unsuccessful due to cell lysis occurring after a few hours of normal growth. To address this issue, we modified the sporulation agar by reducing the concentration of nutrient broth (Difco) from 8 g/L to 2 g/L. In this adjusted medium, the bacteria exhibited an extended growth period, with some sporulation observed near the air interface. Further optimizations, including increasing air space and replacing a portion of the fresh modified sporulation medium with overnight spent medium, enabled the bacterial population to complete the sporulation process, culminating in the release of fully developed spores from the mother cells.

### Phase-bright spore formation precedes ENA protein expression by hours

**Fig. 1:**
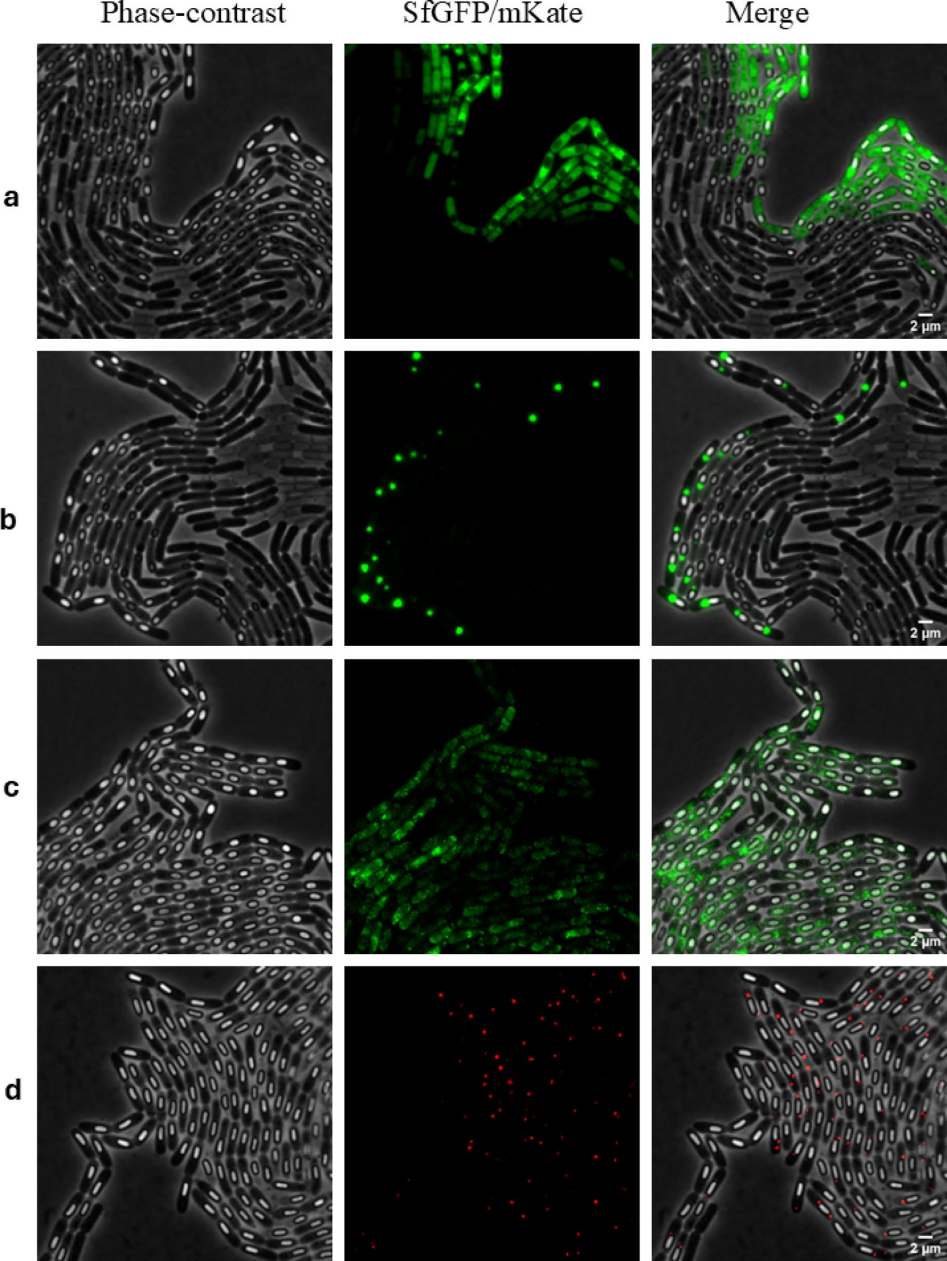
ENA subunit proteins are expressed after phase-bright spores mature. Time-lapse fluorescent images of *B. paranthracis* showing ENA protein expression. (**a**) Ena1A-sfGFP, (**b**) sfGFP-Ena1B, (**c**) Ena1C-sfGFP and (**d**) Ena3A-mkate2. Fluorescence images shown in panel (**a**–**d**) were from chromosomally integrated constructs, whereas panel (**b**) represents expression from a low-copy plasmid (pHT304).

To investigate the temporal dynamics of ENA subunit expression during sporulation, we made constructs encoding fluorescent fusion proteins (sfGFP or mKate2) at the C- or N-termini of ENA subunits. The constructs were either integrated into their native chromosomal loci or expressed from low-copy plasmids under native promoters. When expressed from plasmids, expression was analyzed in both the wildtype strain and relevant *ena* deletion mutants as appropriate. Fluorescent protein-tagged Ena1A, Ena1B, Ena1C, and Ena3A were monitored by time-lapse fluorescence microscopy. Starting from single vegetative cells, images were taken every 10–12 min and followed until spore release from the mother cell. When needed, complementary data were obtained from shake-flask cultures bysampling at 1-h intervals until the sporulation was completed. Under the conditions we used, the first detectable fluorescence signals, indicating the onset of ENA subunit expression, appeared approximately ~ 1–3 h after phase-bright spores became clearly visible. Fluorescence intensity then increased steadily until mature spores were released from the mother cells. Notably, expression of all four *ena* genes was tightly regulated, with no Ena1A, Ena1B, Ena1C or Ena3A detected before the spores attained maximum size and brightness (Fig. 1, Supplementary Fig. S4 and Supplementary Movie S1-4). Occasional exceptions were observed in a few apparently non-sporulating or aberrant cells, where premature expression occurred (Supplementary Fig. S5). Importantly, these rare instances of leaky expression were restricted to strains expressing Ena from the low-copy plasmid pHT304 (Supplementary Fig. S5) and were absent in strains with chromosomally integrated constructs.

### ENA expression peaks at the forespore-mother cell interface during late sporulation phase

Having established the timing of ENA subunit expression during sporulation, we next examined the precise spatial localization and intensity dynamics of each Ena protein. This analysis revealed distinct patterns of fluorescence accumulation on the surface of the developing spore highlighting key differences in the subcellular distribution of Ena1A, Ena1B, Ena1C, and Ena3A during the late stages of spore development. The sections below describe the expression pattern and localization of each Ena protein.

#### Ena1A

**Fig. 2:**
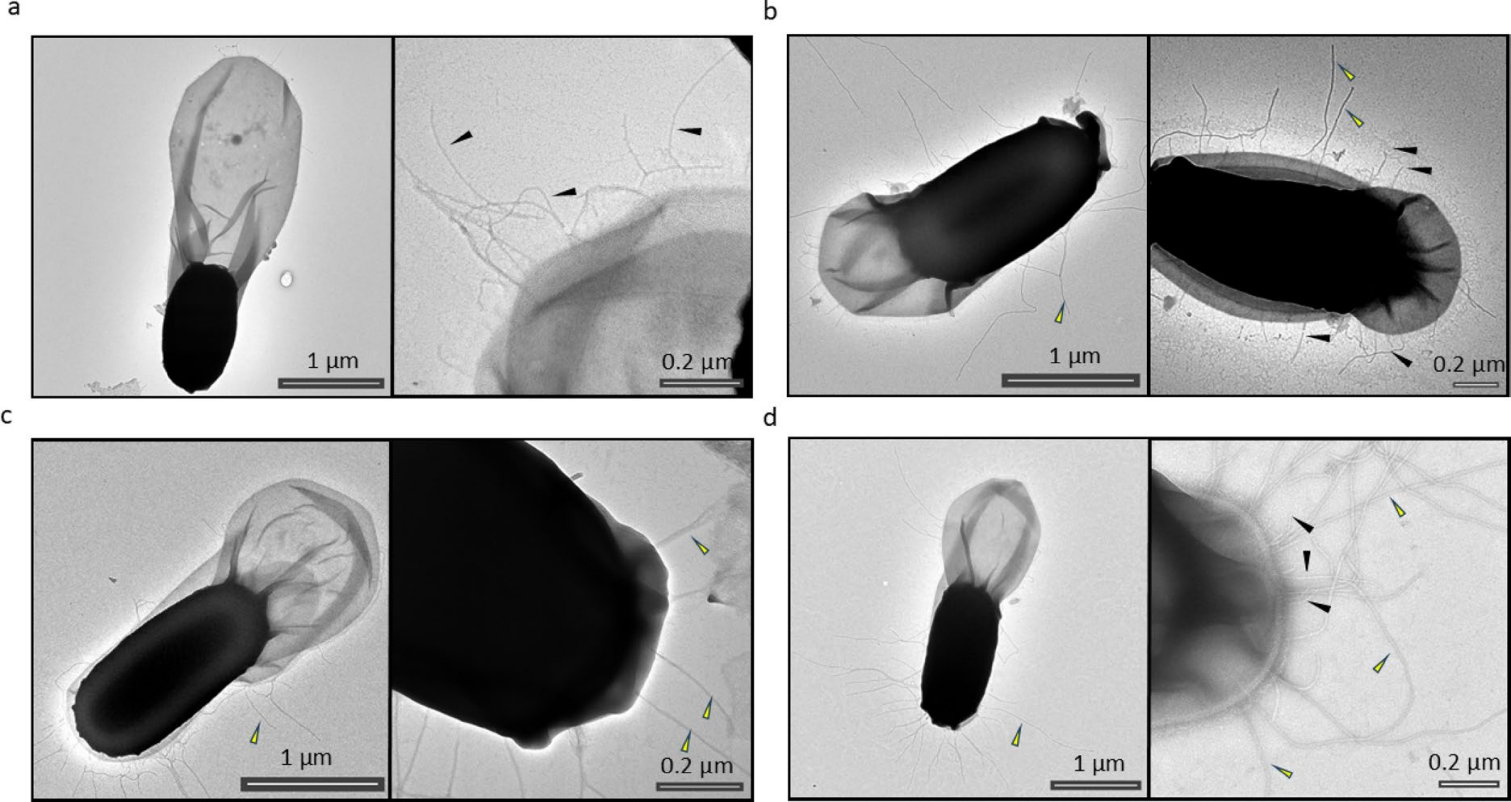
nsTEM micrographs of C-terminal fluorescent protein-tagged constructs integrated at their native *ena* loci in *B. paranthracis*. (**a**) *B. paranthracis ena1A::sfGFP.* (**b**) *B. paranthracis ena1C*::*sfGFP.* (**c**) *B. paranthracis ena3A::mKate2.* (**d**) wildtype *B. paranthracis* (control). Yellow and black arrows indicate S-ENAs and L-ENAs, respectively.

Ena1A was expressed as a C-terminal fusion with *sfGFP* integrated into the chromosome (*B. paranthracis ena1A::sfGFP*). Under the conditions used, Ena1A expression began approximately 1.8 h after the first phase-bright spores were detected, with fluorescence increasing steadily over the following 2.5 h (Fig. 1 and Supplementary Movie S1). Fluorescence was initially distributed throughout the mother cell, followed by a marked accumulation on the developing spore. After spore release, fluorescence remained visible within the exosporial sac (Supplementary Fig. S6). nsTEM examination of these spores revealed an absence of surface displayed S-ENA fibers, suggesting that the sfGFP tag interfered with S-ENA polymerization (Fig. 2a).

#### Ena1B

**Fig. 3:**
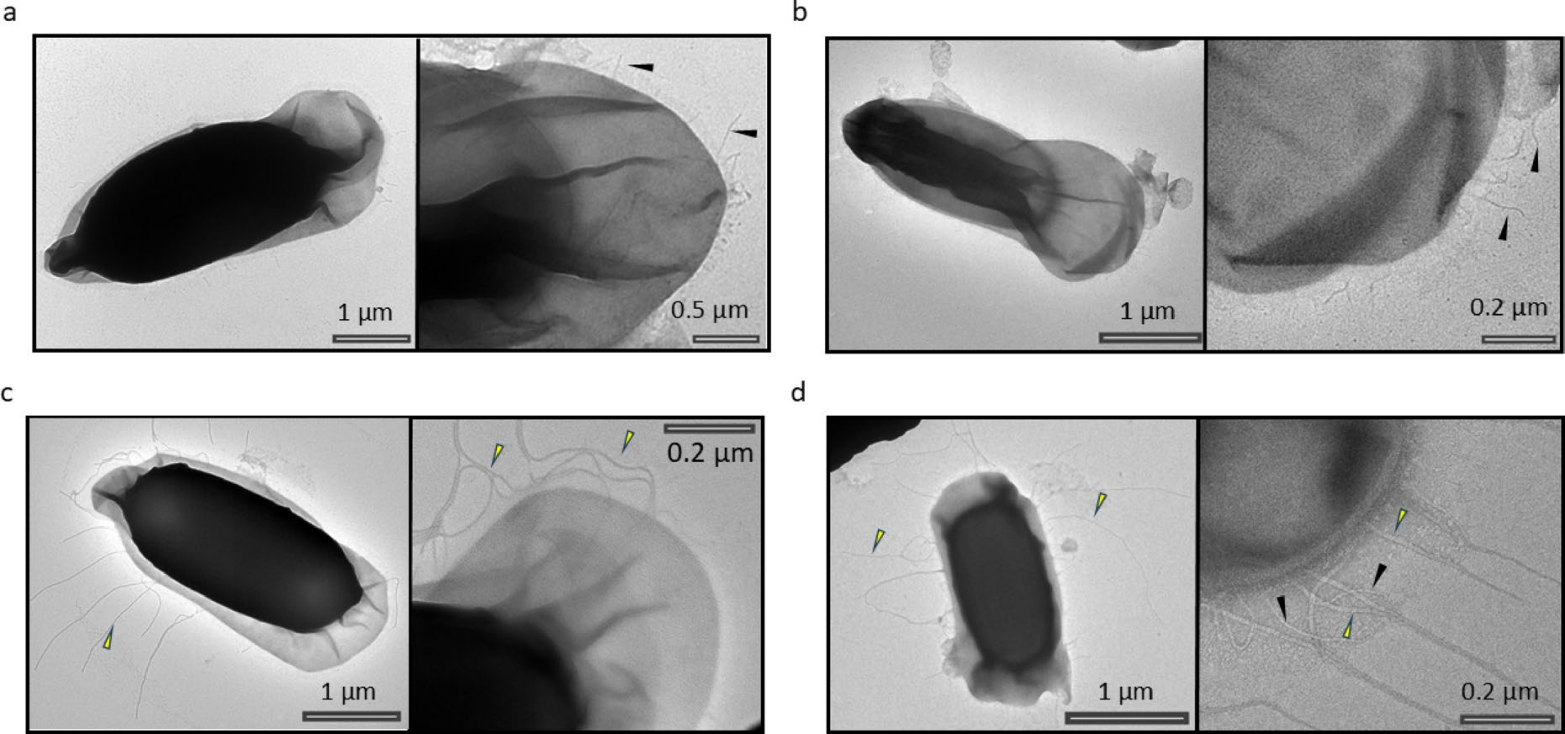
nsTEM micrographs of spores of wildtype *B. paranthracis* transformed with pHT304 expression constructs encoding fluorescent tagged ENA subunits. (**a**) *B. paranthracis*::pHT304-P_*ena1AB*_*-sfGFP-ena1B*. (**b**) *B. paranthracis*::pHT304-P_*ena1AB*_*-ena1B-sfGFP* (**c**) *B. paranthracis*::pHT304-P_*ena3A*_*-mKate2-ena3A*. (**d**) *B. paranthracis*::pHT304 (control). Yellow and black arrows indicate S-ENAs and L-ENAs, respectively.

N-terminally sfGFP tagged Ena1B was expressed from a low-copy plasmid. Under the conditions used, the first GFP signal appeared approximately 1.5 h after detection of the first phase-bright spores. Ena1B expression began predominantly at a single focus near the forespore, oriented toward the mother cell side (Fig. 1). Signal intensity steadily increased over the next 3 h until the spore was released from the mother cell (Supplementary Movie S2). Despite considerable loss of Ena1B during mother cell lysis (Supplementary Movie S2), released spores still retained varying levels of fluorescence (Supplementary Fig. S7). A similar expression pattern was observed when the tag was fused to the C-terminus (Supplementary Fig. S7b). Whereas nearly all released spores carrying the N-terminally tagged Ena1B exhibited fluorescence, only 32% of spores expressing the C-terminally tagged version were fluorescent (over 200 spores examined for each construct; Supplementary Fig. S7c). nsTEM analysis of spores from both N- and C-terminally tagged Ena1B expressed from plasmids revealed an absence of S-ENA fibers (Fig. 3a, b), indicating that the tag interfered with S-ENA assembly even in a wildtype background.

#### Ena1C

To investigate the spatiotemporal expression pattern of Ena1C, we generated fusion proteins in which sfGFP was attached to the C-terminus of Ena1C (chromosomally integrated) or mKate2 was fused to its N-terminus and expressed from a low-copy plasmid. The Ena1C-sfGFP fusion produced diffuse fluorescens foci, predominantly localized near the spore body, which started to appear approximately 2 h after emergence of phase-bright spores (Fig. 1 and Supplementary Movie S3). Notably, fluorescence signals remained associated with the spores after lysis of the mother cells (Supplementary Movie S3 and Supplementary Fig. 8a). Similarly, spores released from cells expressing mKate2-Ena1C from a low-copy plasmid also retained fluorescence (Supplementary Fig. S8b). Interestingly, nsTEM of *B. paranthracis ena1C::sfGFP* spores revealed fewer S-ENA (13 ± 7.4 on average) compared to wildtype spores (20 ± 9), based on examination of 50 spores per group (Figs. 2b and 5e).

#### Ena3A

Ena3A, the major component of the L-ENA fiber^6^, was expressed as a fusion with mKate2, either chromosomally at the C- or plasmid-borne at the N-terminus. In both constructs, expression was evident as a few bright foci on either side of the spore, predominantly adjacent to the mother cell (Fig. 1, Supplementary Movie S4). Expression was first detected at approximately 3.1 h after the appearance of phase-bright spores. The fluorescence signal from Ena3A fused C-terminally with mKate2 diminished considerably upon lysis of the mother cell but was not lost completely (Supplementary Movie S4). The remaining signal was detected in the exosporium of the mature spore (Supplementary Fig. S9a; Supplementary Movie S4). In contrast, an apparent loss of fluorescence was evident upon lysis of the mother cell when the tag was placed at the N-terminus of Ena3A (Supplementary Fig. S9b, c) in the automated time-lapse experiment. To verify this, we analyzed spores (over 200 in each group) from 24-h shake-flask cultures of the two constructs after washing twice with PBS. The results indicate that a subpopulation of spores from both constructs retained detectable fluorescence, with 48% of the C-tagged spores and 41% of the N-tagged Ena3A spores remaining fluorescent. nsTEM analysis of spores expressing chromosomally integrated Ena3A-mKate2 (*B. paranthracis ena3A::mKate2*) or plasmid-expressed Ena3A-mKate2 in the wildtype strain (*B. paranthracis*::pHT304*-Pena3A-mKate2-ena3A*) revealed a complete absence of L-ENAs (Figs. 2c and 3c).

In summary, ENA fusion proteins exhibited distinct temporal and spatial expression patterns during sporulation, with fluorescence signals primarily accumulating at the forespore–mother cell interface. Ena1A and Ena1C localized more broadly around the spore, whereas Ena1B and Ena3A showed highly focused expression. Expression from low-copy plasmid was stronger than from chromosomal integrations, and a substantial fraction of ENA fusion proteins remained unincorporated into spores.

### The expression of S-ENA proteins precedes L-ENA expression

**Fig. 4:**
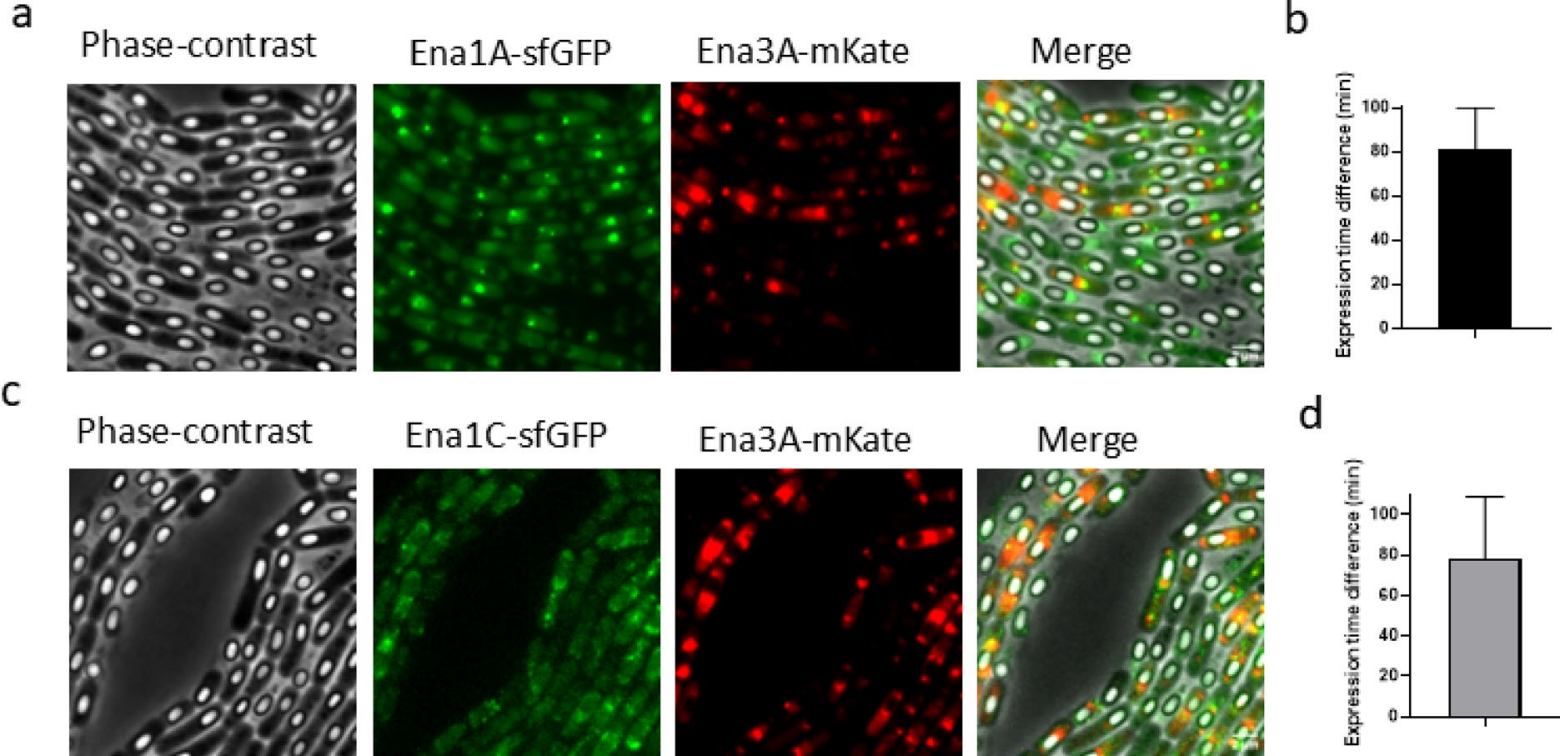
Co-expression of S-and L-ENA subunit proteins. (**a**) Co-expression of Ena1A-sfGFP (chromosome) and mKate2-Ena3A (plasmid-borne). (**b**) Time (min) between detection of Ena1A and detection of Ena3A expression. (**c**) Co-expression of Ena1C-sfGFP (chromosome) and mKate2-Ena3A (plasmid-borne). (**d**) Time (min) between initial detection of Ena1C and detection of Ena3A expression. Error bars in (**b**) and (**d**) are SD.

To determine the relative expression timing of S- and L-ENA subunit proteins, we co-expressed Ena1A fused to sfGFP (chromosomal integration) and Ena3A fused to mKate2 (plasmid-borne), each under its respective native promoter. Time-lapse fluorescence microscopy revealed that Ena1A expression began approximately 80 min before Ena3A (Fig. 4a, b and Supplementary Movie S5). Similarly, co-expression of Ena1C-sfGFP (chromosome) with mKate2-Ena3A (plasmid) showed that Ena1C expression preceded Ena3A by ~ 79 min (Fig. 4c,d and Supplementary Movie S6). Even after accounting for the differences in fluorescent protein maturation time, 13.6 min for sfGFP and 34.4 min for mKate2^24^, the S-ENA proteins Ena1A and Ena1C were expressed approximately 1 h earlier than the L-ENA subunit Ena3A. However, because fluorescent protein maturation kinetics depend on cellular physiology and have not been characterized in the sporulating mother cell, the precise timing of fluorescence appearance, and consequently the relative timing between the two reporters, should be interpreted with caution.

### Ena1C is required for anchoring S-ENA fibers to the spore surface

**Fig. 5:**
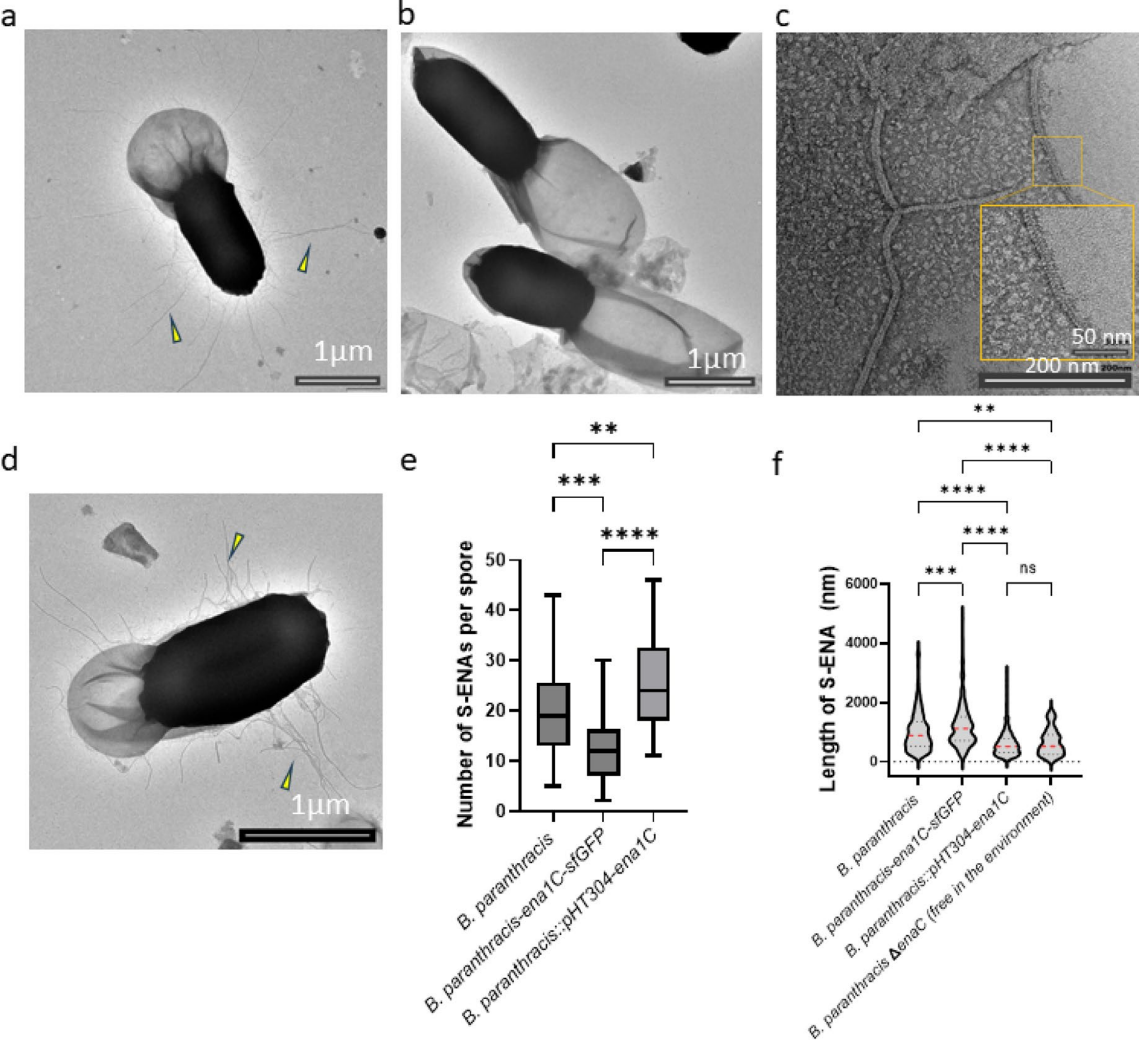
Ena1C plays a role in anchoring S-ENA to the spore. (**a**) nsTEM of *B. paranthracis* wildtype spores. Yellow arrows indicate S-ENAs. (**b**) *B. paranthracis* ∆*ena1C* spores lacking attached S-ENAs. (**c**) nsTEM of free S-ENAs found in culture supernatant of *B. paranthracis* ∆*ena1C.* (**d**) *B. paranthracis*::pHT304-*ena1C* (Ena1C overexpressing strain). (**e**) Box plot showing the effect of *ena1C* on the number of anchored S-ENAs per spore. The horizontal line represents the median, and whiskers denote the minimum and maximum values. (**f**) Violin plot of S-ENA lengths in wildtype spores (*n* = 268), *ena1C-sfGfp* (*n* = 193), Ena1C overexpressing spores (*B. paranthracis*::pHT304-*ena1C*) (*n* = 436) and ∆*ena1C* spores (unattached S-ENAs free in the environment). The median S-ENA length is indicated by a red dashed line, with quartiles in black.

Previously, the study by Pradhan et al.^5^ reported that deletion of *ena1C* in *B. paranthracis* (Fig. 5a) results in spores lacking S-ENA fibers (Fig. 5b), even though Ena1C is not a structural component of the S-ENA fiber stalk. In that study, spores were extensively washed prior to analysis, potentially removing loosely associated material. In light of the hypothesis that Ena1C anchors the S-ENA fiber to the spore coat and given the reduced number of S-ENA observed in spores expressing Ena1C fused to sfGFP (Fig. 5e), we investigated whether detached or loosely associated S-ENA fibers might still be present in spores lacking Ena1C. To this end, we examined unwashed spore preparations of the *B. paranthracis* ∆*ena1C* mutant. Notably, free S-ENAs were observed in close proximity to the ∆*ena1C* spores (Fig. 5c), without being anchored to the spore surface, suggesting that Ena1C is potentially involved in S-ENA attachment to the spore. The reduction in surface-attached S-ENA fibers observed in Ena1C-sfGFP spores indicates that the C-terminal fusion interferes with Ena1C function in anchoring the S-ENAs to the sub-exosporial layer (Figs. 2b and 5e). We further hypothesized that if Ena1C indeed has role in anchoring S-ENA, overexpression of Ena1C could lead to more, but shorter number of surface-attached S-ENA fibers. Accordingly, we transformed the wildtype *B. paranthracis* strain with a low-copy plasmid expressing untagged Ena1C under its native promoter. Consistent with the proposed role of Ena1C in anchoring S-ENAs, these spores exhibited a significantly higher number of S-ENAs per spore (Fig. 5d and e). Indeed, the S-ENAs in Ena1C overexpressing strain were also relatively shorter than those in the wildtype strain (Fig. 5f), but comparable in length to the free S-ENAs observed in the *ena1C* knockout strain. Notably, the median length of free ENAs in the *ena1C* knockout (Fig. 5b) and overexpressing strains (Fig. 5d) were nearly identical (521 nm and 518 nm, respectively), while the wildtype and C-terminal sfGFP-Ena1C strain had median length of 882 and 1118 nm, respectively (Fig. 5f).

### Cryo-EM analysis of Ena1C

**Fig. 6:**
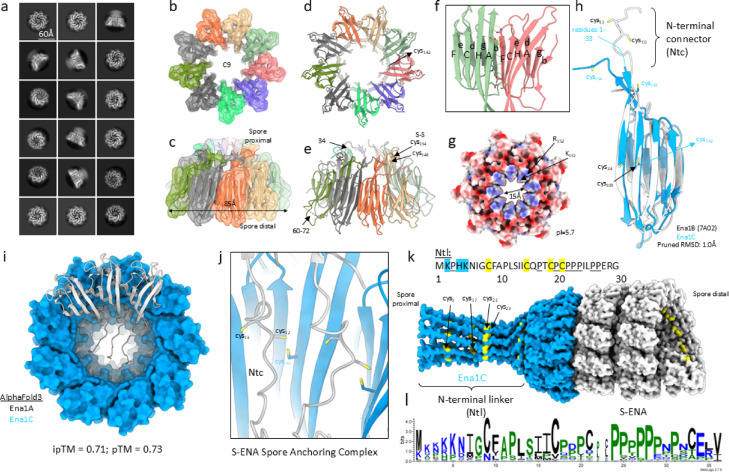
Cryo-EM structure of Ena1C. (**a**) 2D class averages of rec-Ena1C; (**b**) and (**c**) Top and tilt views of the reconstructed Ena1C cryo-EM volume color coded according to chain id. The ring diameter of Ena1C is 85Å; (**d**) and (**e**) Top and tilt views of the Ena1C nonamer model shown in cartoon. (**d**) 9 copies of cys_142_ reside at the interior lumen of the Ena1C ring; (**e**) Cysteines 148 and 154 participate in an inter-chain disulfide bridge, repeated 9-fold, covalently reinforcing the Ena1C ring; (**f**) Lateral pairwise docking of Ena1C protomers is mediated via β-sheet augmentation (the docking interface is strand F and b of two neighboring subunits). Strands of the foremost β-sheet are shown in uppercase letters (FCHA), and strands of the rear β-sheet are shown in lowercase (edgb); (**g**) electrostatic surface potential colored according to local charge: residues K_151_ and R_152_ form a positively charged lining of the C-terminal funnel which has an exit diameter of 15Å; (**h**) An Ena1C protomer superimposed onto the major subunit Ena1B of the S-ENA helix (PDB: 7A02) showing excellent structural alignment characterized by a pruned Ca root-mean squared deviation (RMSD) of 1.0Å. Crucially, the position of Cys_109_ of Ena1B is conserved in Ena1C (Cys_142_); (**i**) AlphaFold3 prediction of a 9:3 Ena1C/Ena1A multimer (ipTM:0.71; pTM: 0.73); (**j**) Zoom-in of the putative docking of the Ena1A N-terminal connectors in the lumen of Ena1C ring forming a putative S-S bond between cys_142_ (Ena1C) and cys_12_; (**k**) Proposed model for the S-ENA spore anchoring complex. (**l**) Weblogo representation of the multiple sequence alignment of the N-terminal linker (NTL) of Ena1C.

To further elucidate the role of Ena1C in the coupling mechanism of S-ENA spores, we proceeded with a cryo-EM study of recombinantly produced Ena1C. A codon-optimized coding sequence of the full-length Ena1C was cloned into the plasmid pET28a and transformed into *E. coli* strain BL21 (DE3). To facilitate affinity chromatography purification, we cloned a 6xHis tag at the 5ꞌ end of the Ena1C open reading frame and performed expression tests. As AlphaFold modelling predicted the first 36 N-terminal residues of Ena1C to be disordered, those residues were removed. The resulting construct used in cryo-EM was named 6xHis-Ena1C (∆1–36). While SDS-PAGE analysis confirmed successful expression of Ena1C, the protein accumulated in inclusion bodies and was therefore purified via Ni-NTA under denaturing conditions (i.e. in the presence of 8 M urea). SDS-PAGE analysis of the purified protein revealed a predominant SDS-stable species > 100 kDa, consistent with AlphaFold3 modelling, which predicted nonameric assemblies (Supplementary Fig. S11). To further remove any misfolded, aggregated species and/or monomeric Ena1C subunits, we pooled the Ena1C containing fractions, performed a buffer exchange to non-denaturing conditions (25 mM potassium phosphate buffer pH 7.5), and passed the sample over a size-exclusion column. Fractions enriched towards the > 100 kDa species were concentrated to 1.0 mg/mL, applied to a freshly glow-discharged holey-carbon grid, and plunge-frozen in liquid ethane. A high resolution cryo-EM dataset was collected on a JEOL CryoARM 300 microscope and processed using a standard single particle processing method in CryoSPARC. 2D classification identified that particles were predominantly aligned in top-view orientations (Fig. 6a), although a minor fraction of tilted and side-view classes could be resolved after extensive 2D classification. The top view classes revealed a ring-like structure with clear nine-fold symmetry, which was in agreement with AlphaFold predictions and the presence of high-molecular weight species in SDS-PAGE. No top-view classes were observed with other than 9-fold symmetry (e.g. no 8- or 10-fold rings), indicating that recombinant Ena1C exclusively forms nonameric ringlike structures. Crucially, no fiber-like species were observed in any of the 2D class averages or in the raw micrographs, confirming the expectation that Ena1C does not form ENA-like fiber structures. We obtained a 3D reconstruction of the Ena1C ring at 2.9 Å global resolution (0.143 FSC criterion) using Homogenous Refinement in CryoSPARC v 4.7.1, enforcing global C9 symmetry. It is important to note that as a result of preferential orientation (i.e. top-view dominated), that the resulting volume is quite anisotropic and that the reported 2.9 Å resolution is only applicable along the Z-axis, and does not reflect the map resolution along other viewing directions. The conical FSC Area Ratio (cFAR) is 0.16, where a cFAR value smaller than 0.5 is

used as a rule of thumb to identify map anisotropy (Supplementary Fig. S12). Attempts to lower the map anisotropy via the CryoSPARC jobs “Rebalance 2D Classes” or “Rebalance Orientations” did not lead to improvements in map quality. To ameliorate the limitations in map quality and enhance map interpretability, we performed map post-processing using EMReady^23^. The resulting volume (Fig. 6b,c) resolved the individual Ena1C subunits with secondary structure elements clearly visible and allowed for a rigid body docking of an AlphaFold3 Ena1C nonamer model (Fig. 6d,e). No interpretable density was observed for residues 60 to 72 which are predicted to form a loop connecting strands A and b (Fig. 6e). The Ena1C protomers form an 85Å open ring structure that is covalently stabilized by 9 reoccurring intermolecular disulfide bridges between cysteines 148 and 154 (Fig. 6e). Neighboring subunits bilaterally dock (Fig. 6f) onto each other with their respective solvent exposed strands F (of sheet FCHA of subunit *i*) and b (of sheet edgb of subunit *j*), in a process of β-sheet augmentation (effectively yielding one extended β-sheet composed of strands FCHAedgb). This mode of protomer docking is reminiscent of the subunit contacts in the S-ENA and L-ENA fibers. The Ena1C ring terminates in a funnel-like opening that is positively charged (residues K_151_ and R_152_) and has a diameter of 15Å (Fig. 6g). A single Ena1C subunit constitutes an 8-stranded β-sandwich domain (Fig. 6h) with a 33 amino acid N-terminal extension (not resolved in the cryo-EM volume, but modelled with AF3; vide infra) that shares clear structural homology (pruned Cα RMSD is 1.0Å) with Ena1B (and by extension with Ena1A)^5^, the two major subunits of the S-ENA fiber (we only show the superposition with Ena1B for clarity). Interestingly, the position of cys_109_ of Ena1B (and the corresponding cys_118_ in Ena1A) is conserved in Ena1C through cys_142_. This is relevant because cys_109_ of Ena1B is involved in the covalent coupling of the N-terminal connector of subunit i + 9, which leads to a structural reinforcement of the S-ENA fiber in the axial direction. Based on that structural similarity, and the observation that ∆*ena1C* spores are deficient in spore-attached S-ENA fibers (Fig. 5b), we reasoned that Cys_142_ of Ena1C could function as a coupling agent to bind the S-ENA fiber to the spore surface using a tethering mechanism reliant on S-S bond formation between cys142 (Ena1C) and cys10 or cys11 located at the N-terminal connector (Ntc) of Ena1B, or the equivalent cys12/13 of Ena1A. Indeed, transforming *ena1C* knockout strain with an expression plasmid encoding Ena1C in which Cys 142 was substituted with serine failed to complement the ∆*ena1C* phenotype, which is characterized by the absence of S-ENAs (Fig. 5b; Supplementary Fig. S16). This also fits with our topological understanding of the S-ENA fibers. Namely, in a previous study, we identified the fiber polarity and showed that the Ntc is directed towards the spore surface, making the solvent accessible thiol groups in the Ntc logical points of coupling. To explore that hypothesis further, we produced an AlphaFold3 prediction of a 9:3 Ena1C: Ena1A multimer (Fig. 6i; Supplementary Fig. S13). AF3 predicts that the 3 Ena1A subunits dock onto the solvent exposed rim of the Ena1C ring, with their respective Ntcs pointing downwards into the ring lumen, positioning cys_12_ to engage in disulfide bond formation with cys_142_ of Ena1C. To some extent, this Ena1C/Ena1A complex mimics the homotypic Ntc-mediated axial contacts that exist natively in the S-ENA fiber, with one notable distinction, however. Namely, there is a clear symmetry mismatch between Ena1C (i.e. C9), and S-ENA (helical: rise = 3.2Å, twist=−31.03°). The helical rise forces Ena1A subunits to gradually be positioned away from the Ena1C ring surface (at 3.2Å increments, or 25.6Å in total for 9 subunits). This means that it is unlikely that 9 Ena1A protomers can reasonably be expected to dock and couple their respective Ntcs, but that only the leading 3–4 subunits might be able to do so, depending on the flexibility and extensibility of the Ntc. By combining the Ena1C and S-ENA cryo-EM structures, with the mode of docking predicted by AF3, we can generate an integrated model for the S-ENA spore anchoring complex (Fig. 6j,k). In this model we also include the predicted 1–33 amino acids (hereafter referred to as the N-terminal linker) of Ena1C which form an N-terminal extension to the ring structure. It is important to note that the pLDDT values of these residues are low, meaning that the confidence of the structural prediction is low. Whether or not these low pLDDT values follow from intrinsic flexibility or disorder of the N-terminal linker (NTL) region remains unclear at this point. Interestingly, there are 4 cysteine residues in the NTL. We speculate that these might be required for the coupling of the S-ENA spore anchoring complex to an as of yet unidentified receptor on the spore coat surface. We also note that there are numerous proline residues (nine in total). If we perform a blastp search using Ena1C as a query and generate a weblogo derived from a multiple sequence alignment of the NTL sequences, we find that both the cys and pro residues are strongly conserved (Fig. 6l). Interestingly, the weblogo has a region of 6 consecutive proline residues. Poly-proline sequences typically form one of two types of helices (PPI or PPII), which tend to be rigid and extended structures. We hypothesize that proline-rich character of the NTL could contribute to the overall rigidity and extension of the NTL, essentially priming it for docking onto (and perhaps penetrating in) the spore coat.

## Discussion

For half a century, ENAs have remained enigmatic protein fibers, their structural secrets concealed by extraordinary resilience. Only with recent advances in cryo-EM have researchers begun to unveil their intricate architecture and decipher the genetic blueprints behind their formation^5–7^. This, in turn, has opened the door to exploring their potential functional roles in *Bacillus* species^4,10^. A recent study showed that ENAs enable spores to adhere strongly to abiotic surfaces, such as stainless steel and polypropylene^11^. Adhered spores pose a challenge in food industries as they can withstand cleaning-in-place (CIP) procedures allowing microorganisms to gradually build up on food production equipment and surfaces over time^25,26^. While spores adhered to polypropylene show increased tolerance to alkaline treatments^27^, those attached to surfaces such as stainless steel and rubber exhibit enhanced heat resistance compared to their planktonic counterparts^28,29^. Given the significance of spore-forming species in public health, the food industry, agriculture and biowarfare, as well as the widespread presence of ENAs in both *Bacillus* and *Clostridium* species^5–7^, a comprehensive understanding of ENA expression and regulatory mechanisms could inform the development of effective strategies to reduce or prevent spore contamination, improve CIP processes in industrial settings, or enhance adhesion in beneficial applications, such as the use of *B. thuringiensis* as a biopesticide.

Owing to the recent discovery of *ena* genes, little is known about the spatiotemporal expression of ENAs, the relative expression of S-and L-ENAs, or the specific functions of ENA-associated genes, particularly *ena1C*, in S-ENA biogenesis. In this study, we employed time-lapse fluorescence microscopy, nsTEM and cryo-EM to address these questions. Our findings revealed that expression of both S- and L-ENA subunits from the chromosome is tightly regulated and occurs exclusively after phase-bright spores have formed and matured inside the mother cell. This conclusion is supported by the observation that spores reached their maximum size and brightness at the time of ENA expression, indicating that ENAs form at a later stage of the sporulation phase. Notably, the earliest detection of ENA expression occurred 1.5 h after phase-bright spores became visible. The strict regulation of ENA expressions and the absence of expression of either of the four *ena* genes examined prior to phase-bright spore formation, suggests that ENAs do not play a role during the vegetative growth of *Bacillus* spp. As S- and L-ENAs emerge from the spore coat and exosporium, respectively^5^, and their subunits are expressed late in the mother cell during sporulation (Fig. 1), it is likely that the late sporulation sigma factor σ^K^ regulates *ena* transcription. Indeed, in *B. thuringiensis*, σ^K^ regulates the expression of hair-like nap proteins and basal layer proteins, among others^30^, whereas in *B. subtilis*, it controls the production of the coat proteins, coat-associated polysaccharides, and precursors essential for spore cortex synthesis^31^.

The expression and translocation of ENA subunit proteins from the mother cell to the surface of the developing spore are supported by a sequence of observations: fluorescence distributed in the mother cell, even at sites distant from the forespore compartment (Fig. 1 and Supplementary Fig. S10); a subsequent increase in fluorescence intensity near the mother cell-forespore interface (Supplementary Movie S1-4); and ultimately, the retention of fluorescent proteins associated with the released spores after mother cell lysis (Supplementary Figs. S6-S9). These findings suggest that ENA proteins expressed in the mother cell must be translocated to the developing spore for assembly. Known mother-forespore channels such as SpoIIIAH-SpoIIQ^32,33^, and SpoVV/SpoVA complexes^34^ mediate transport during earlier sporulation stages, but they do not appear to contribute to ENA deposition, as S-ENAs and L-ENAs are expressed during late sporulation phase and localize to the coat and exosporium, respectively. This implies that S-ENA and L-ENA subunits are deposited in a manner analogous to canonical coat and exosporium proteins, respectively.

In some constructs, a significant portion of the expressed ENA proteins failed to associate with the spore, resulting in an abrupt loss of fluorescence following mother cell lysis (e.g. Supplementary Movie S2). Interestingly, analysis of spores released from cells carrying these constructs, after washing with PBS, revealed that a subset retained detectable levels of fluorescence (Supplementary Fig. S7). The heterogeneity in fluorescence retention among released spores within the same population could be attributed to incomplete formation or rupture of the exosporium, as previously reported in *B. paranthracis*^5^. Importantly, prior studies showed that the assembly of recombinant His_6_-Ena1B was arrested at a single turn, likely due to steric hindrance from the affinity tag. Given that sfGFP and mKate2 are substantially larger than a His_6_ tag, these fluorescent proteins are expected to impose an even greater steric hindrance. This bulkiness may interfere with proper folding or assembly of ENA proteins and hinder their translocation and/or deposition leading to the loss of the respective S- or the L-ENA fibers at the spore surface (Figs. 2 and 3).

As noted, S-ENA fibers emerge from the spore coat^5^, whereas L-ENAs are tethered to the exosporium^6^. Since spore coat morphogenesis precedes exosporium formation^35–37^, S-ENA expression would be expected to initiate earlier. Supporting this, our findings show that the S-ENA proteins Ena1A, Ena1B and Ena1C are expressed approximately 1 h before Ena3A (Fig. 4b, d). Given that ENAs are expressed only after phase-bright spores have fully developed (Fig. 1 and Supplementary Movie S1-6), a key question is whether this time window allows S-ENAs to be incorporated into the spore coat. This apparent discrepancy can be explained by the sequential nature of coat assembly. McKenney and Eichenberger demonstrated that coat formation is a highly regulated process, with proteins recruited in distinct temporal classes^37^. Notably, Class VI proteins are recruited exclusively after the phase-bright stage, similar to the expression pattern of S-ENAs^37^. This suggests that S-ENA integration likely follows a late-stage coat assembly pathway.

Ena1C has been the least characterized of the ENA proteins studied. Previous work showed that *ena1C* expression in *B. paranthracis* increases during sporulation phase, and that its deletion leads to spores lacking S-ENA fibers^5^. Immunogold TEM analysis of ex vivo S-ENA fibers further indicated that Ena1C is not a structural component of these fibers^5^. Given its requirement for S-ENA formation but absence from the main fiber structure, it was hypothesized that Ena1C functions in S-ENA fiber assembly and/or anchoring to the spore surface. Our current findings show that unwashed spore preparations of *B. paranthracis* ∆*ena1C* contain assembled S-ENA in the surrounding environment (Fig. 5C), but these fibers are not anchored to the spore, suggesting that Ena1C is dispensable for fiber assembly but essential for anchoring. Accordingly, our cryo-EM analysis of recombinant Ena1C revealed that it forms a nonameric ring-like structure (Fig. 6). In contrast to Ena1B^5^ and Ena3A^6^, however, Ena1C does not assemble into fibers. We propose that Ena1C rings associate with the outermost layer of the spore coat, where they act as a preferential nucleation site for S-ENA formation. Consequently, overexpression of Ena1C is expected to increase the number of spore-bound Ena1C rings and as the finite pool of Ena1A/B subunits becomes distributed across a greater number of nucleation sites, the average S-ENA length is consequently reduced. This model is consistent with the phenotypes observed in the Ena1C-overexpressing strain (Fig. 5e, f). Furthermore, C-terminal sfGFP tag on Ena1C did not abolish docking but significantly reduced the number of attached S-ENAs compared to the wildtype (Fig. 5e). Notably, the fewer attached S-ENAs in this strain were substantially longer than those of the wildtype (median 1118 nm versus 882 nm; Fig. 5f), which is again consistent with the proposed model. This observation suggests that the fusion protein exhibits impaired nonamerization or reduced efficiency in docking onto the spore coat. Supporting this, nsTEM images of unwashed *B. paranthracis ena1C–sfGFP* spores revealed numerous unanchored S-ENA fibers, indicating weak anchoring (Supplementary Fig. S14) rather than fragmentation, as S-ENAs have been shown to resist breakage up to at least 250 pN^4^. In our previous study on L-ENA, we demonstrated that ExsL tethers L-ENA to the exosporium^6^. This prompted us to consider potential homology between Ena1C and ExsL. However, similarity between the two proteins is limited to the DUF3992 domain at the C-terminus of ExsL^6^. Notably, Ena1C lacks the N-terminal CotZ domain of ExsL, which mediates its integration into the ExsY sheets.

In summary, we show that S- and L-ENA subunit expressions are tightly regulated and restricted to the late stages of spore maturation, consistent with their spore-specific functions. S-ENA subunits are expressed earlier and incorporated into the spore coat, whereas L-ENA appears later and anchors to the exosporium, underscoring their distinct roles and localization patterns. Importantly, we identify Ena1C as the missing link that connects S-ENA to the spore coat. Together, these findings provide new insights into the spatiotemporal regulation of ENA expression and fiber assembly, advancing our understanding of ENA biogenesis and offering a framework for future efforts to modulate ENA function in industrial and applied settings. This study could have been further strengthened by including an additional class VI coat protein and an exosporium protein to enable more direct temporal comparisons with S-and L-ENA subunit proteins, respectively. Looking ahead, identifying interacting partners of ExsL and Ena1C will be key to elucidating the molecular mechanisms of ENA anchoring, and may open new opportunities to engineer spore surface architectures for both fundamental biological studies and biotechnological applications.

## Supplementary Information

Below is the link to the electronic supplementary material.

Supplementary Movie 1.

Supplementary Movie 2.

Supplementary Movie 3.

Supplementary Movie 4.

Supplementary Movie 5.

Supplementary Movie 6.

Supplementary Material.

Supplementary Movie Information.

## Data Availability

The datasets generated during and/or analysed during the current study are available from the corresponding author(s) on reasonable request.
