# Supplementary material for: Spatiotemporal expression of endospore appendages and cryo-EM insights into Ena1C-mediated S-ENA anchoring in Bacillus paranthracis
Source: Sci Rep. 2026 Feb 3;16:7122. doi: 10.1038/s41598-026-38321-0 (PMC12921279; doi:10.1038/s41598-026-38321-0)
Supplement: Supplementary file 7 — Supplementary Material. [file 41598_2026_38321_MOESM7_ESM.pdf]

Supplementary Materials

Spatiotemporal Expression of Endospore Appendages and Cryo-EM Insights into Ena1C-Mediated S-ENA Anchoring in *Bacillus paranthracis*

Ephrem Debebe Zegeye[1, *], Mike Sleutel[2, 3], Unni Lise Jonsmoen[1], Jingqi Chen[4, †], Luiza P. Morawska[4], Yohannes Beyene Mekonnen[1], Oscar P. Kuipers[4], Han Remaut[2, 3] and Marina Aspholm[1, *]

[1]Department of Paraclinical Sciences, Faculty of Veterinary Medicine, Norwegian University of Life Sciences (NMBU), 1433 Ås, Norway. [2]Structural Biology Brussels, Vrije Universiteit Brussel, Brussels, Belgium. [3]Structural and Molecular Microbiology, Structural Biology Research Center, VIB, Brussels, Belgium. [4]Molecular Genetics Group, Groningen Biomolecular Sciences and Biotechnology Institute, University of Groningen, Groningen, The Netherlands. † Current affiliation: Department of Chemistry and the Carl R. Woese Institute for Genomic Biology, University of Illinois at Urbana-Champaign, Urbana, USA.

*Correspondence: marina.aspholm@nmbu.no; ephrem.debebe.zegeye@nmbu.no

Supplementary Table S1. List of plasmid constructs used in this study.

Name	Description/purpose	Source
pMAD-I-SceI	Shuttle vector for making gene replacement constructs	1
pMAD-I-SceI-*ena1A-sfGFP*	Generating *ena1A-sfGFP* single crossover construct	This study
pMAD-I-SceI-*ena1C-sfGFP*	Generating *ena1C-sfGFP* single crossover construct	This study
pMAD-I-SceI-*ena3A-mKate2*	Generating *ena3A-mKate2* single crossover construct	This study
pBKJ223	Encodes the I-SceI enzyme, which introduces a double-stranded break to promote double crossover	1
pHT304	Low copy number plasmid for expression	2
pHT304-P_{ena1AB}-*sfGFP-ena1B*	Expression of N-terminal sfGFP tagged Ena1B from plasmid (native promoter)	This study
pHT304-P_{ena1AB}-*ena1B-sfGFP*	Expression of C-terminal sfGFP tagged Ena1B from plasmid (native promoter)	This study
pHT304-P_{ena3A}-*mKate2-ena3A*	Expression of N-terminal mKate2 tagged Ena3A from plasmid (native promoter)	This study
pHT304-P_{ena3A}-*mKate2-ena1C*	Expression of N-terminal tagged Ena1C tagged with mKate2 via the N-terminus (*ena3A* promoter)	This study
pHT304-P_{ena1C}-*ena1C*	Overexpression of Ena1C in wildtype *B. paranthracis*	3
pHT304-P_{ena1C}-*ena1C* (C142S)	Complementing Δena1C strain	This study
pET28a-His_6-*ena1C*	Recombinant expression of His_6-Ena1C for cryo-EM analysis	This study
pET28a-His_6-*ena1C* (Δ1-36)	Recombinant expression of truncated His_6-Ena1C (Δ1-36) for cryo-EM analysis	This study

Supplementary Table S2. List of primers used in sequencing plasmid and *B. paranthracis* constructs.

**Primer name/code**	**Primer sequences (5 '→3')**	**Source**
pMAD-I-SceI primers (694/695)	CCATCAGACGGTTCGATCTT/ TCCCTGATGGTCGTCATCT	3
pHT304 primers (1541/1542)	TTTATGCTTCCGGCTCGTAT/GGAGAAAATACCGCATCAGG	3
ena1A (2366/2367)	ACGCTTTGTACCGGGAGTTC/ GTTGCAGCTGCTGTCCAATC	This study
ena1C (2368/2369)	AGACTCAGGTGGAGGGACAA/ CACATCGGCCATGCTGTTAC	This study
ena3A (2241/2242)	GGTTGGAGCTGCCTTAACAA/TGAGGGGTCACCATATCAAAA	This study

Supplementary Table S3. Amino acid sequences of plasmid-expressed fluorescent-tagged and non-fluorescent Ena proteins (GFP linker underlined).

Construct	Amino acid sequence	Expression plasmid
mKate2-Ena3A	MSELIKENMHMKLYMEGTVNNHHFKCTSEGEGKPYEGTQTMRIKAVEGG<br>PLPFAFDILATSFMYGSKTFINHTQGIPDFFKQSFPEGFTWERVTTYEDGGV<br>LTATQDTSLQDGCLIYNVKIRGVNFPSNGPVMQKKTLGWEASTETLYPAD<br>GGLEGRADMALKLVGGGHLICNLKTTYRSKKPAKNLKMPGVYYVDRRLE<br>RIKEAYKETYVEQHEVAVARYCDLPSKLGHRGSGGGGSLAQIGNCCTEQL<br>CCVNDAVCCTIILDDTGGTALPIWDDATTFVINGTIMVENNGTVGVGPTAA<br>LTVNGTAVGGFVVAPGECRSITMNDINSIAIVGAGTGTSSVKISFSINYKF*	pHT304
sfGFP-EnaB	MSKGEELFTGVVPILVELDGDVNGHKFSVRGEGEGDATDGKLTLKFICTT<br>GKLPVPWPTLVTTLTYGVQCFSRYPDHMKRHDFFKSAMPEGYVQERTISF<br>KDDGTYKTRAEVKFEGDTLVNRIELKGIDFKEDGNILGHKLEYNFNSHNV<br>YITADKQKNGIKANFKIRHNVEDGSVQLADHYQQNTPIGDGPVLLPDNHY<br>LSTQSVLSKDPNEKRDHMVLLEFVTAAGITHGMDELYKGSGGGGSGNCST<br>NLSCCANGQKTIVQDKVCIDWTAAATAAIIYADNISQDIYASGYLKVDTGT<br>GPVTIVFYSGGVTGTAVETIVVATGSSASFTVRRFDTVTILGTAAAETGEFC<br>MTIRYTLS*	pHT304
EnaB-sfGFP	MSKGEELFTGVVPILVELDGDVNGHKFSVRGEGEGDATDGKLTLKFICTT<br>GKLPVPWPTLVTTLTYGVQCFSRYPDHMKRHDFFKSAMPEGYVQERTISF<br>KDDGTYKTRAEVKFEGDTLVNRIELKGIDFKEDGNILGHKLEYNFNSHNV<br>YITADKQKNGIKANFKIRHNVEDGSVQLADHYQQNTPIGDGPVLLPDNHY<br>LSTQSVLSKDPNEKRDHMVLLEFVTAAGITHGMDELYKGSGGGGSGNCST<br>NLSCCANGQKTIVQDKVCIDWTAAATAAIIYADNISQDIYASGYLKVDTGT<br>GPVTIVFYSGGVTGTAVETIVVATGSSASFTVRRFDTVTILGTAAAETGEFC<br>MTIRYTLS*	pHT304
His_6-TEV-Ena1C (Full length)	MHHHHHHSSGENLYFQGKPHKNIGCFAPLSIICQPTCPCPPPILPPERGDAE<br>LVTNEFAGDILISNDFIPISQKQLKQTNTTVNIWKNDGIVSLSGTISIYNNRN<br>STNALSIQIISSTTNTFTALPGNTISYTGFDLQSVSVIDIPSDPSIYIEGRYCFQ<br>LTYCKSKRDCL*	pET28a
His_6-TEV-Ena1C (Δ1-36)	MHHHHHHSLVTNEFAGDILISNDFIPISQKQLKQTNTTVNIWKNDGIVSLSG<br>TISIYNNRNSTNALSIQIISSTTNTFTALPGNTISYTGFDLQSVSVIDIPSDPSIY<br>IEGRYCFQLTYCKSKRDCL*	pET28a

Supplementary Table S4. Cryo-EM data of Ena1C.

Parameter	Ena1C
Voltage (kV)	300
Electron exposure (e^{-} $Å^{-2}$)	60
Pixel size (Å)	0.695
Particle images (n)	412,005
Point group	C9
Conical FSC Area Ratio (cFAR)*	0.21
Sampling Compensation Factor (SCF)**	0.693
Map:map FSC (0.143)	3.0
Model:map FSC (0.5)	6.9
d_{99}	3.80
Ramachandran Favored (%)	98.06
Ramachandran Outliers (%)	0.0
RSCC	0.47
Clash score	15.28
Bonds RMSD, length (Å)	0.012
Bonds RMSD, angles (°)	1.358
**Deposition ID**	
PDB (model)	9T3P
EMDB (map)	EMD-55504

*a cFAR value of 0.5 serves as a reasonable threshold for the presence, or lack thereof, of preferred orientation.

**SCF measures how the orientation distribution affects the spectral signal-to-noise ratio. SCF has a range of [0, 1] with higher numbers indicating better sampling distributions. A value of 0.81 (8/pi^2) corresponds to a `uniform side view` case.

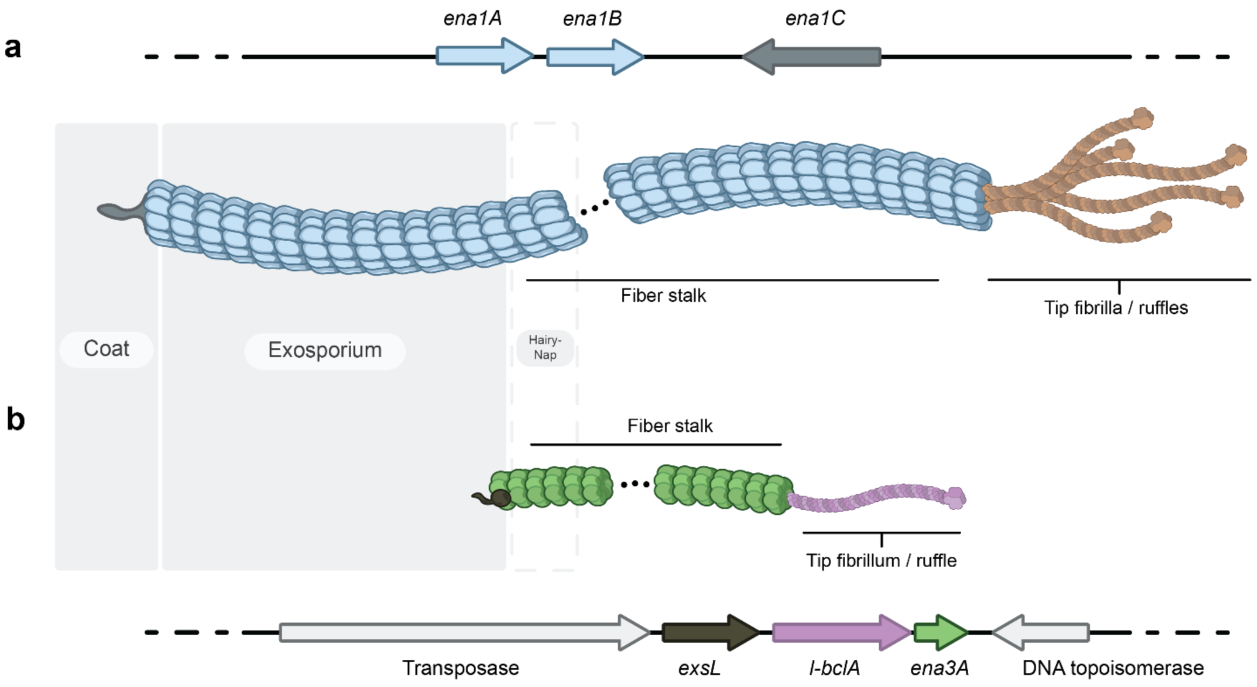

Supplementary Fig. S1. Illustration of genes and structure of (a) the S-ENA and (b) L-ENA fibers. The S-ENA is encoded by the *ena1A* and *ena1B* genes (blue). The L-ENA fiber is encoded by the *ena3* gene cluster.

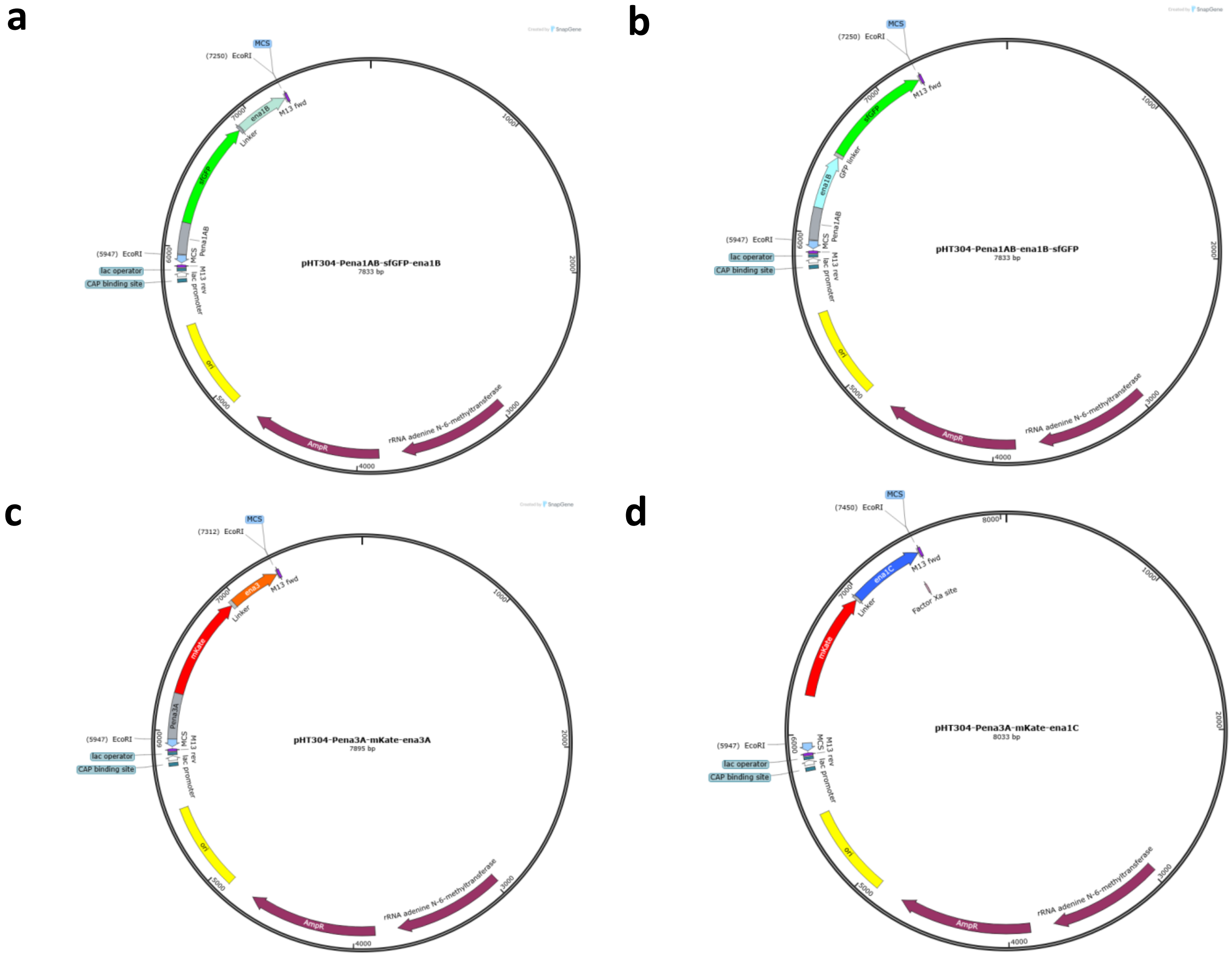

Supplementary Fig. S2. Plasmid maps of low-copy ENA expression constructs using pHT304 plasmid. (**a**) pHT304-*P*$_{ena1AB}$-*sfGFP-ena1B* (N-terminally tagged Ena1B). (**b**) pHT304-*P*$_{ena1AB}$-*ena1B-sfGFP* (C-terminally tagged Ena1B) (**c**) pHT304-*P*$_{ena3A}$-*mKate2-ena3A*. (**d**) pHT304-*P*$_{ena3A}$-*mKate2-ena1C* (N-terminally tagged Ena1C).

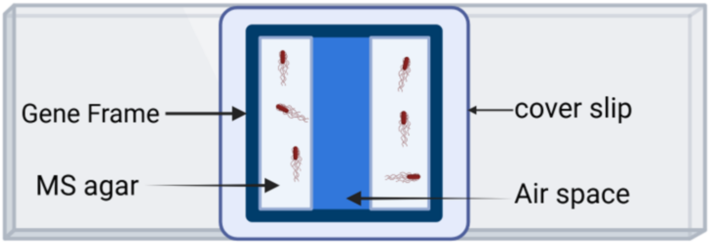

Supplementary Fig. S3. Schematic illustration of a microscopic slide agar preparation used for automated time-lapse fluorescence microscopy. The agar pad consists of modified sporulation (MS) medium supplemented with 1.5% agar. Gene Frame (1.5 × 1.6 cm; Thermo Scientific #AB0577). The Figure was created using BioRender.

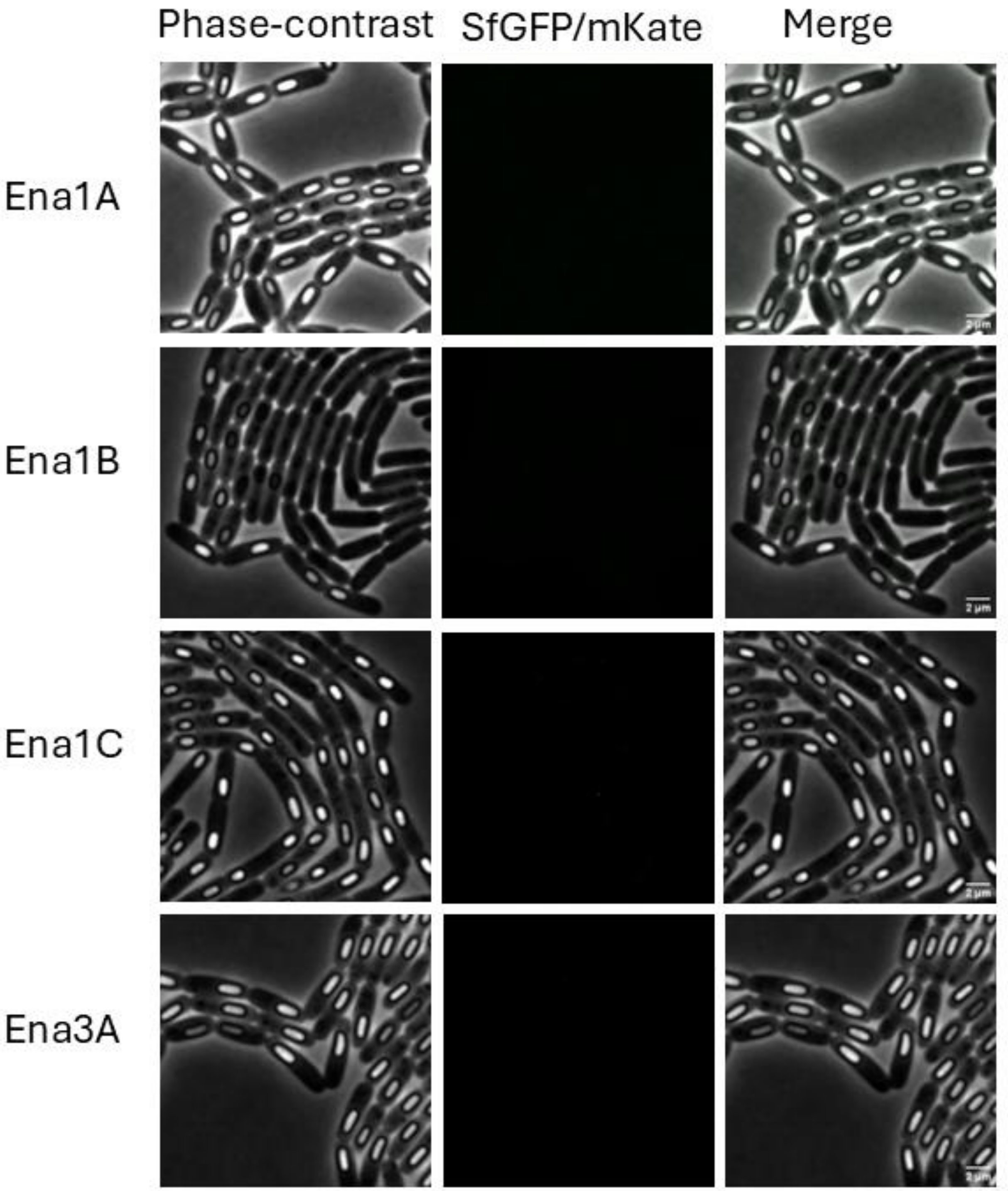

Supplementary Fig. S4. **ENAs are not expressed before phase-bright spores have developed**. Time-lapse fluorescent images of *B. paranthracis*. Ena1A, Ena1C and Ena3A were expressed from chromosome, while Ena1B was expressed from plasmid. The images were captured approximately 10-30 min prior to the initial detection of ENA expression.

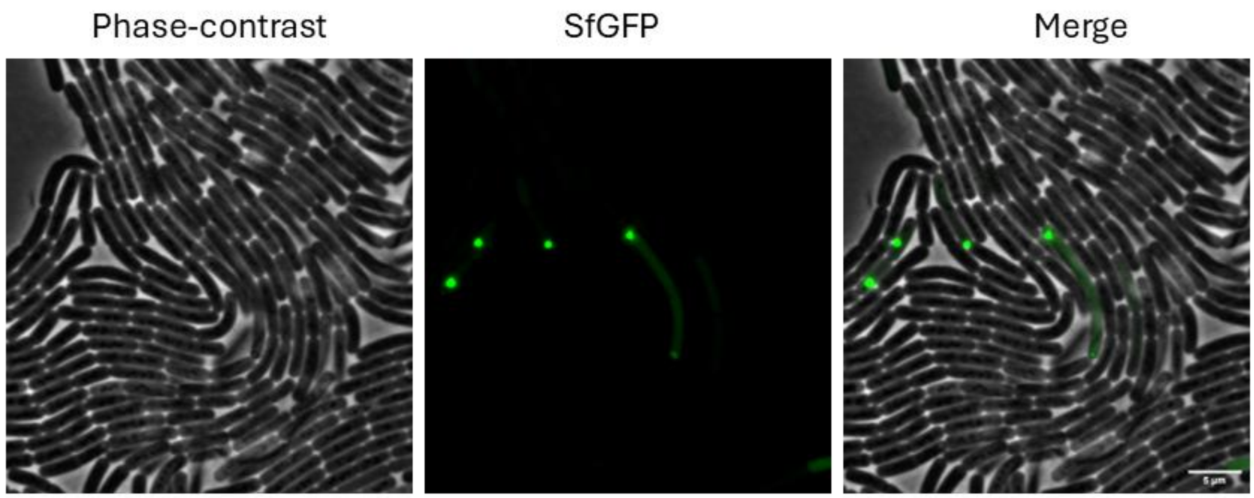

Supplementary Fig. S5. Leaky expression of Ena1B observed in a subset of non-sporulating *B. paranthracis* cells. The strain harbored the plasmid construct pHT304-P_{ena1AB}-*sfGFP-ena1B* in the *B. paranthracis* background.

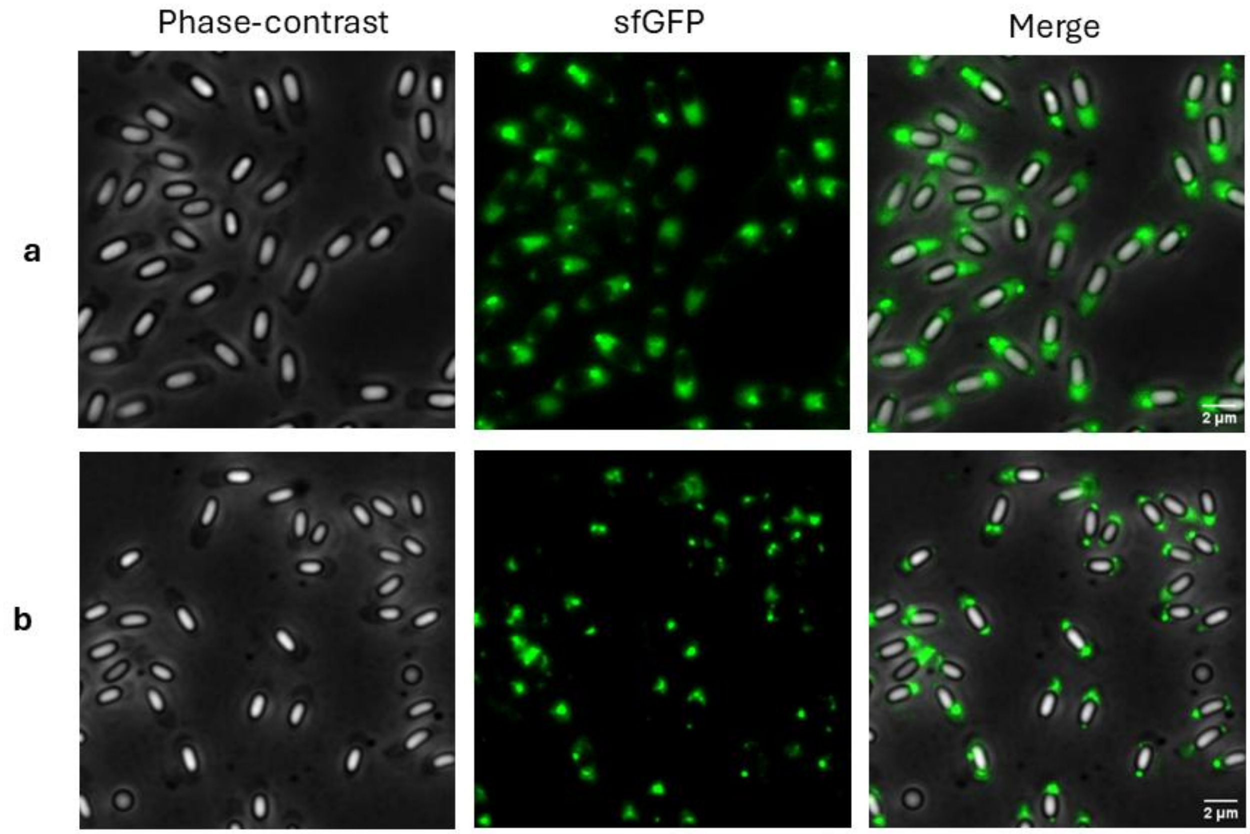

Supplementary Fig. S6. Fluorescence microscopy of *B. paranthracis ena1A::sfGFP*. (**a**) Image from a time-lapse microscopy experiment showing released spores still retaining fluorescent proteins. (**b**) Image from a shake-flask culture 24 h post-inoculation.

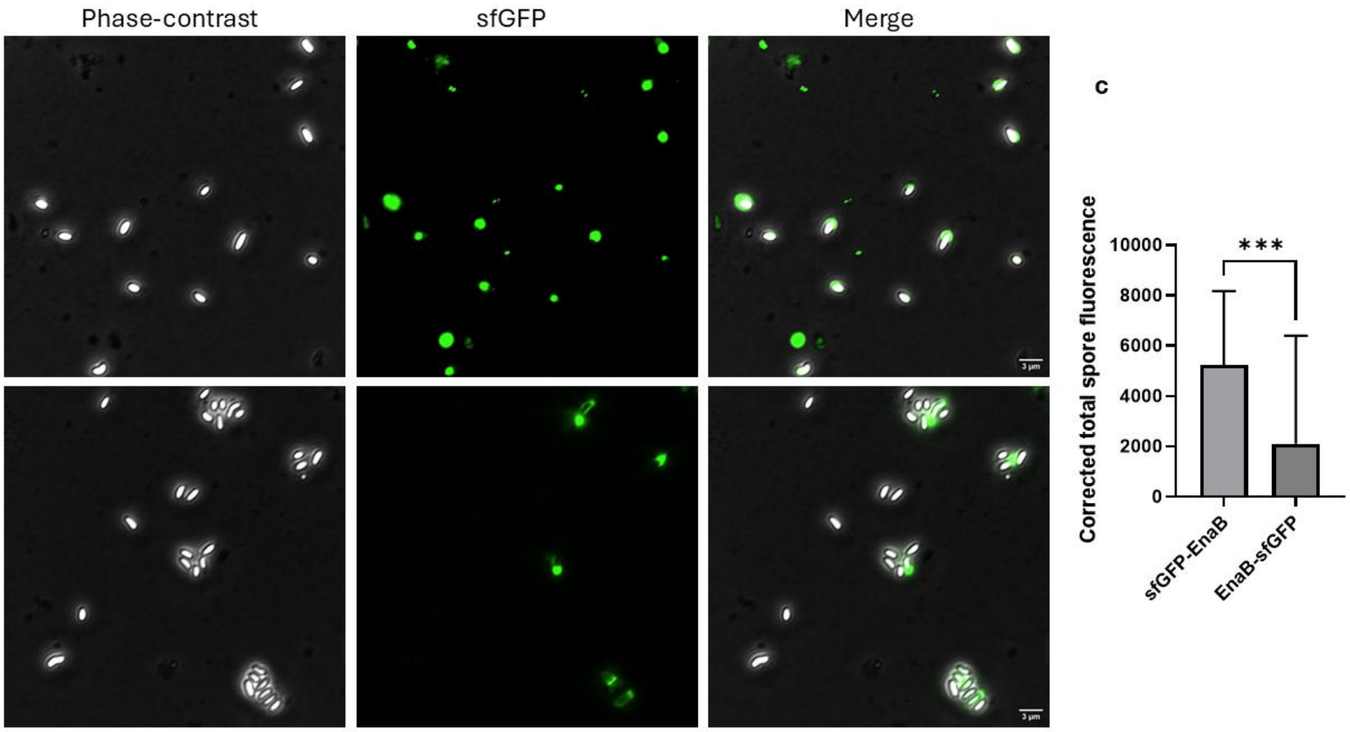

Supplementary Fig. S7. SfGFP-tagged Ena1B localizes to spores when fused at either the N- or C-terminus. Images were acquired from 24-h shake-flask cultures after PBS washing. (**a**) *B. paranthracis::*pHT304-P_{ena1AB}-*sfGFP-ena1B*. (**b**) *B. paranthracis::*pHT304-P_{ena1AB}-*ena1B-sfGFP*. (**c**) N-terminally sfGFP-tagged Ena1B exhibits higher incorporation into spores compared to C-terminally tagged Ena1B.

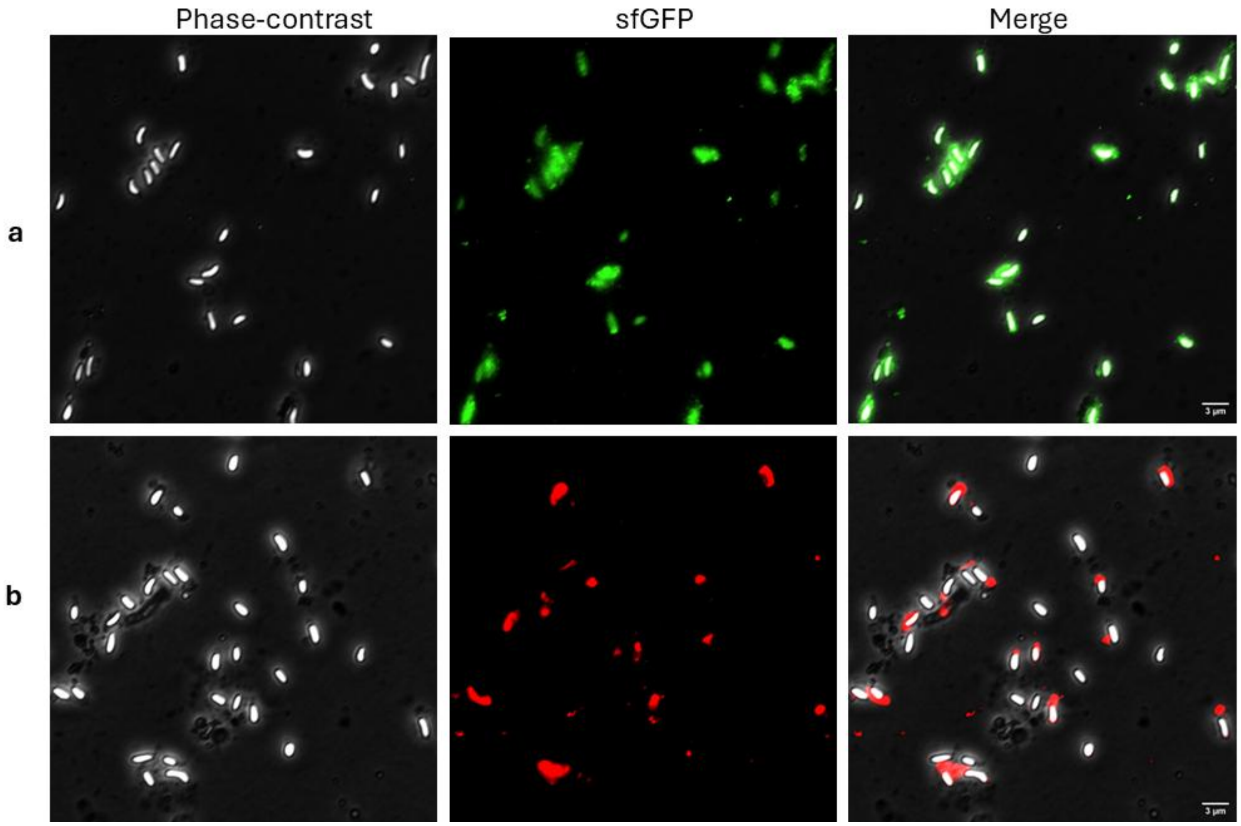

Supplementary Fig. S8. Fluorescently tagged Ena1C localizes to spores when fused at the C-terminus (a) or N-terminus (b). **(a)** *B. paranthracis ena1C::sfGFP*. **(b)** *B. paranthracis*::pHT304-P_{ena3A}-*mKate2-ena1C*. Images were acquired from 24-h cultures after PBS washing.

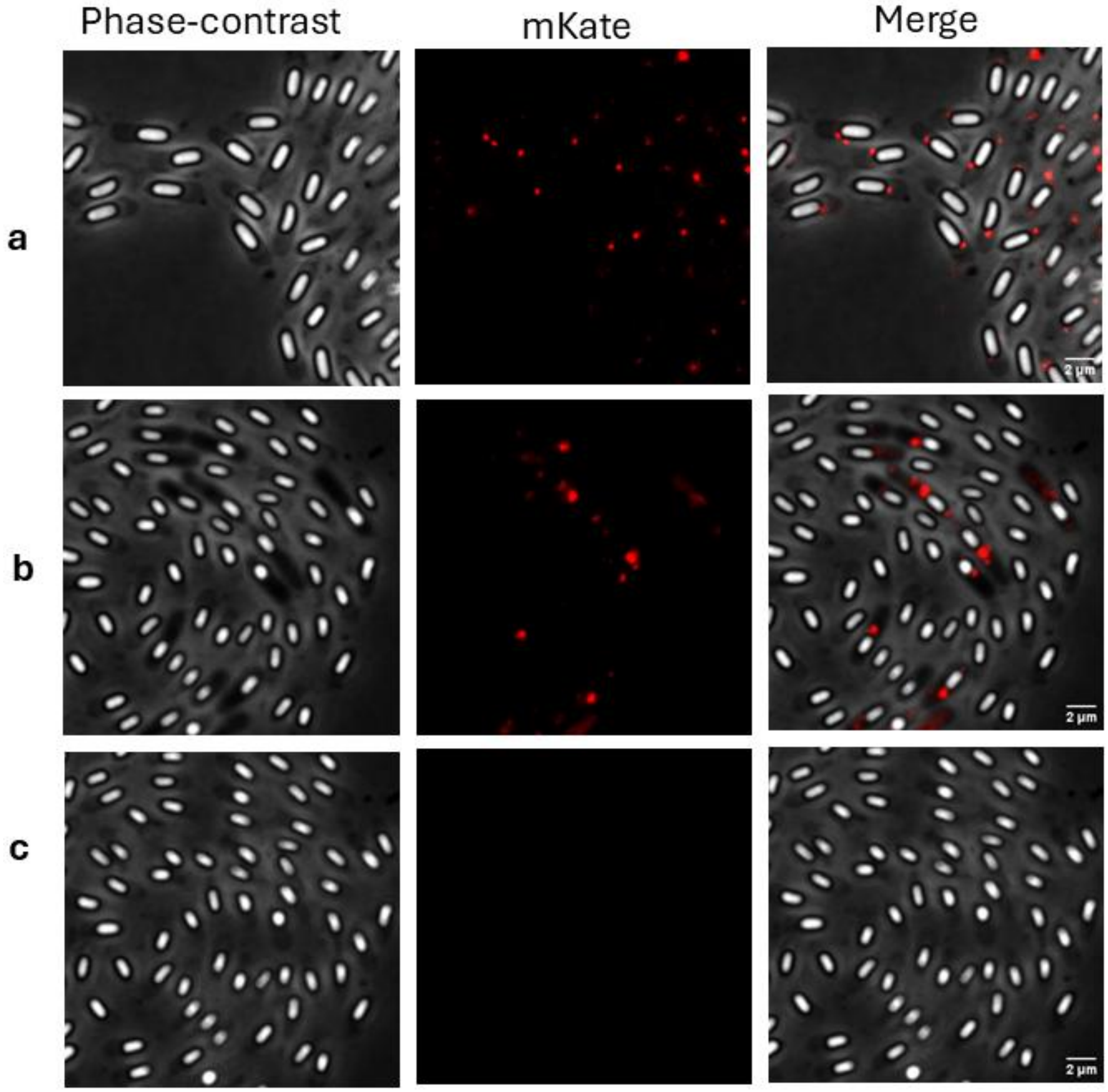

Supplementary Fig. S9. C-terminally mKate2-tagged Ena3A is more efficiently incorporated into the spore than N-terminally tagged Ena3A. (**a**) *B. paranthracis ena3A*::*mKate2* (22 h post inoculation). (**b**) *B. paranthracis*::pHT304-P_{ena3A}-*mKate2-ena3A* (15.5 h post inoculation), and (**c**) *B. paranthracis*::pHT304-P_{ena3A}-*mKate2-ena3A* (18.5 h post inoculation).

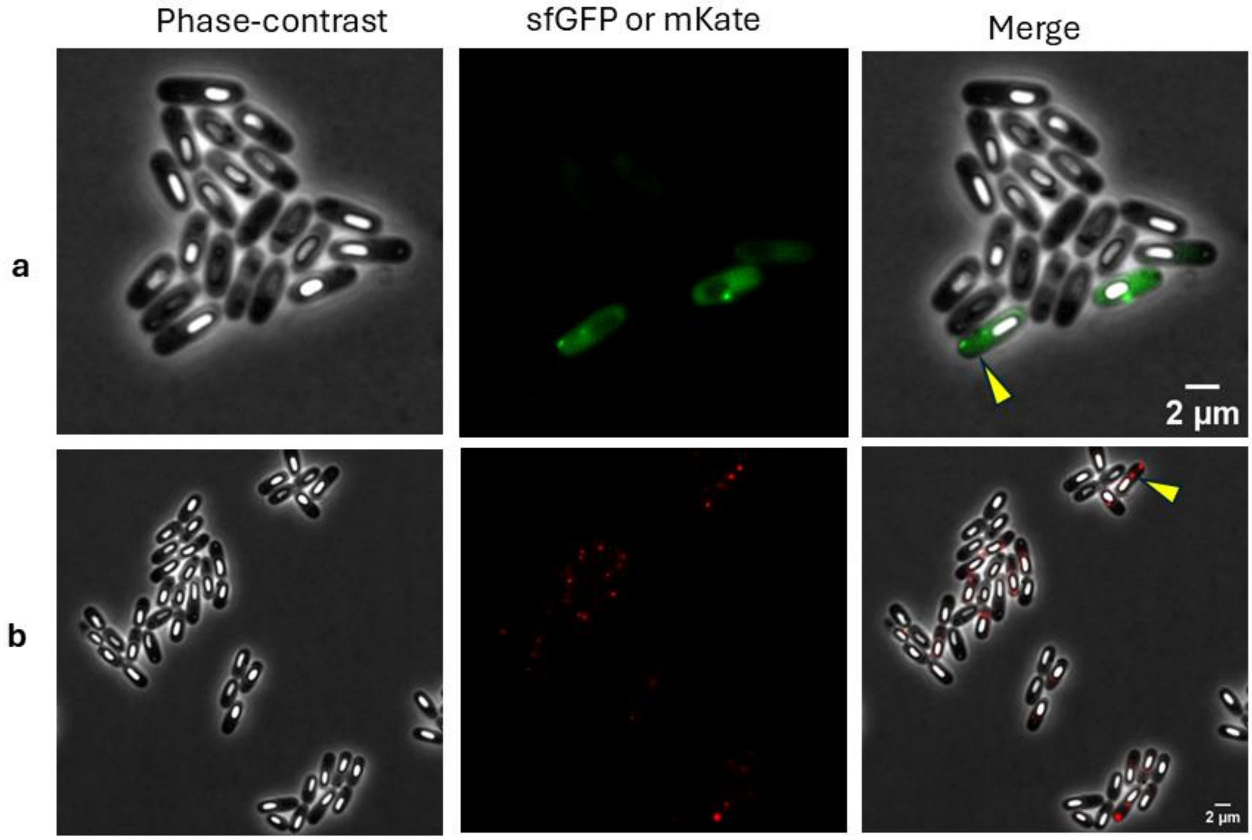

Supplementary Fig. S10. Expression of ENA subunits in the mother cell. Images were captured 9–10 h post-inoculation under flask culture conditions. Yellow arrows indicate distinct expression foci localized within the mother cell, positioned away from the forespore. (**a**) *B. paranthracis ena1A::sfGFP*. (**b**) *B. paranthracis ena3A::mKate2*.

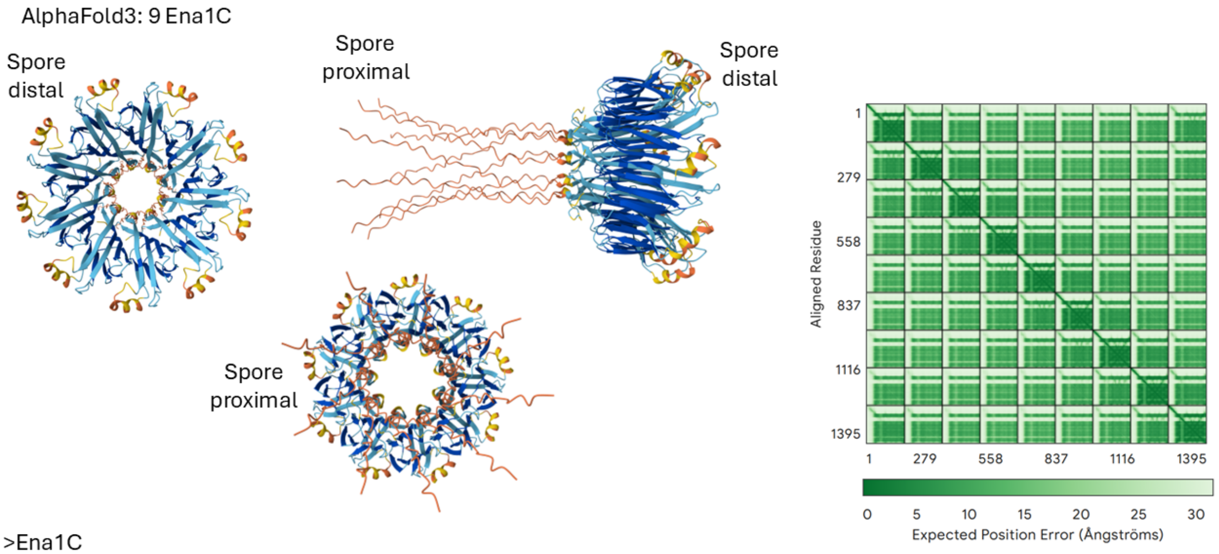

>Ena1C
MKPHKNIGCFAPLSIICQPTCPCPPPILPPERGDAELVTNEFAGDILISNDFIPISQKQLK
QTNTTVNIWKNDGIVSLSGTISIYNNRNSTNALSIQIISSTTNTFTALPGNTISYTGFDLQS
VSVIDIPSDPSIYIEGRYCFQLTYCKSKRDCL

ipTM = 0.66; pTM = 0.67

Supplementary Fig. S11. AlphaFold3 prediction of a Ena1C nonameric complex. Structures are colored according to pLDDT values, and the predicted aligned error (PAE) plot is shown on the right.

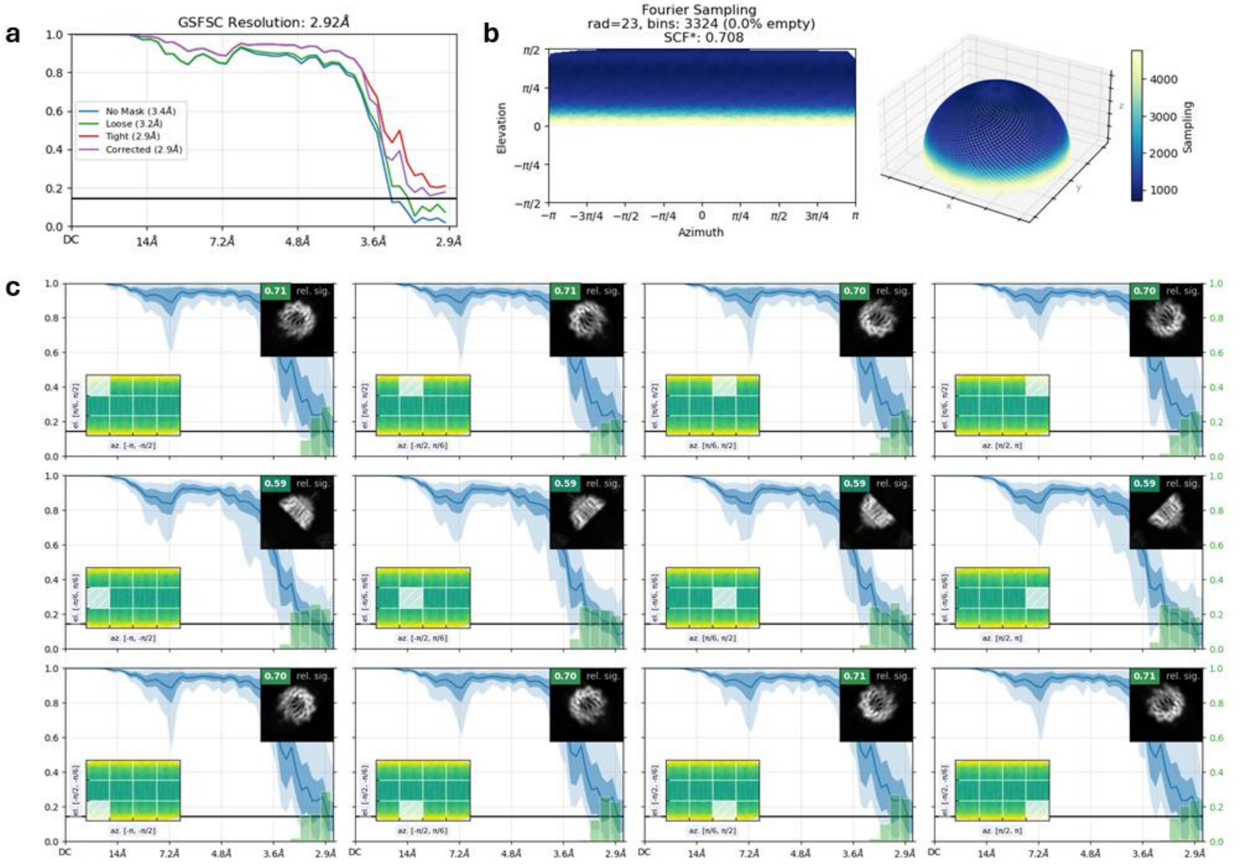

Supplementary Fig. S12. **Orientation Diagnostics of the Ena1C cryo-EM reconstruction**. (**a**) The global resolution estimated by the map:map Fourier Shell Correlation; (**b**) The Sampling Compensation Factor[4] assesses the degree to which certain Fourier voxels are under sampled by the set of particle alignments. Baldwin and Lyumkis argue that values above 0.81 generally indicate good sampling; (**c**) The Average Relative Signal Amount within Azimuth-Elevation Viewing Regions.

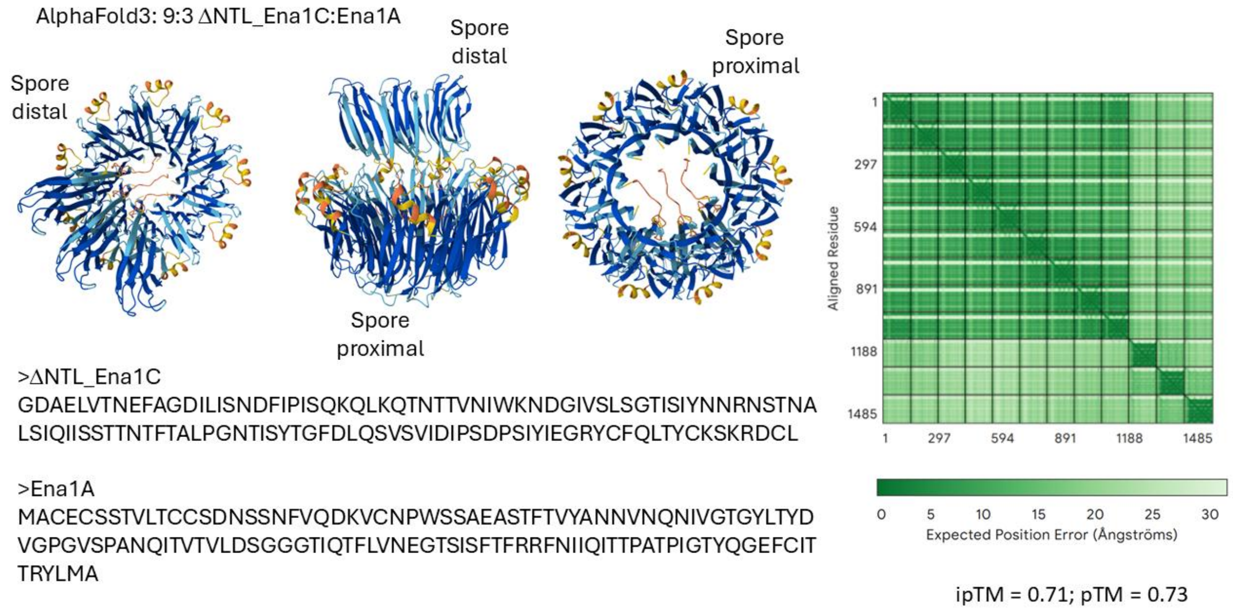

Supplementary Fig. S13. **AlphaFold3 prediction of the 9:3 DNTL_Ena1C:Ena1A complex**. Structures are colored according to pLDDT values, and the predicted aligned error (PAE) plot is shown on the right.

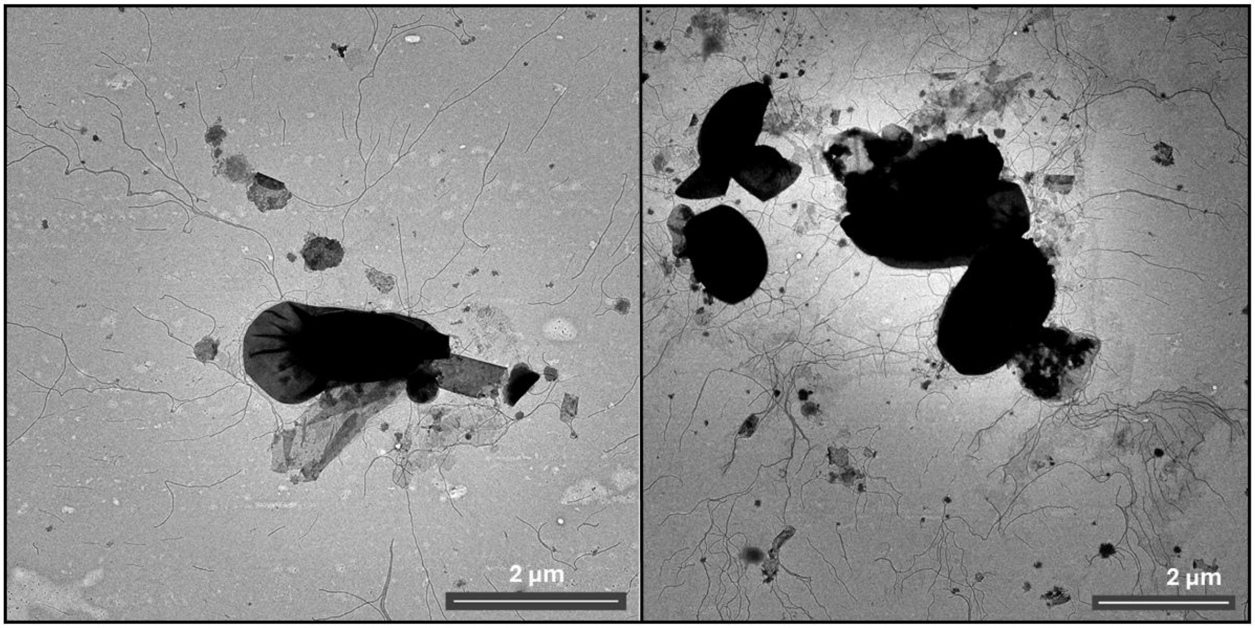

Supplementary Fig. S14. nsTEM of unwashed spore preparation of *B. paranthracis ena1C-sfGFP* showing numerous unattached S-ENAs in the surrounding environment.

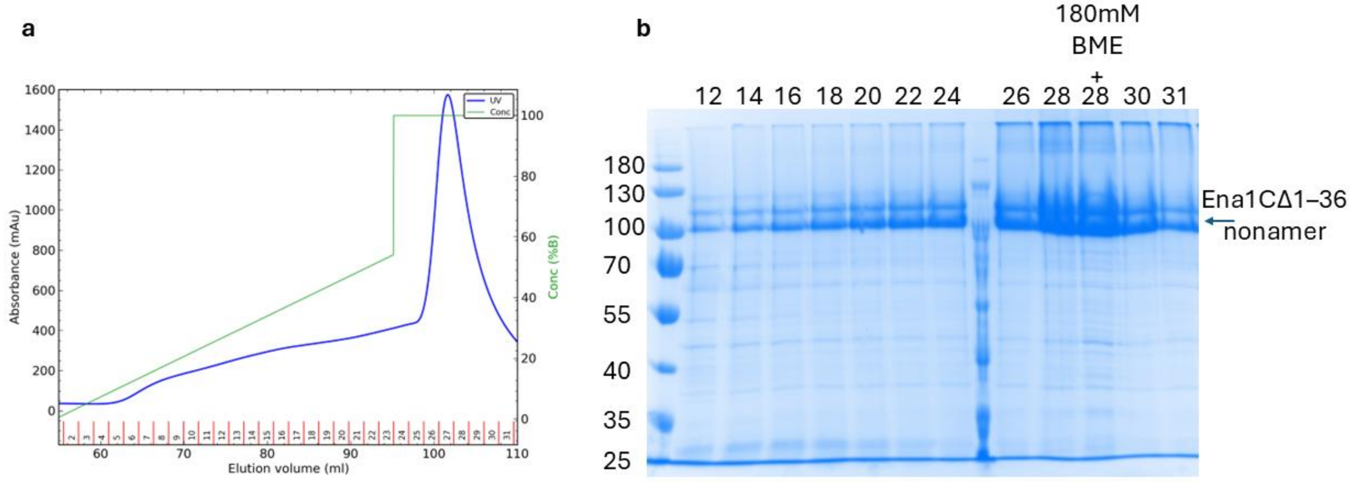

Supplementary Fig. S15. (a) HisTrap affinity chromatography elution profile of recombinant 6xHis-Ena1C (Δ1–36). The blue trace corresponds to the UV 280nm absorption, and the green trace represents the gradient elution profile (i.e. percent buffer B); (b) SDS-PAGE purification of recombinant 6xHis-Ena1C Δ1–36. The lanes correspond to the different fractions of the histrap gradient elution. The samples were loaded onto the SDS-PAGE under oxidative conditions, apart from fraction 28 which was supplemented with 180 mM β-mercaptoethanol. Fractions 26-30 were pooled and concentrated for cryo-EM sample preparation.

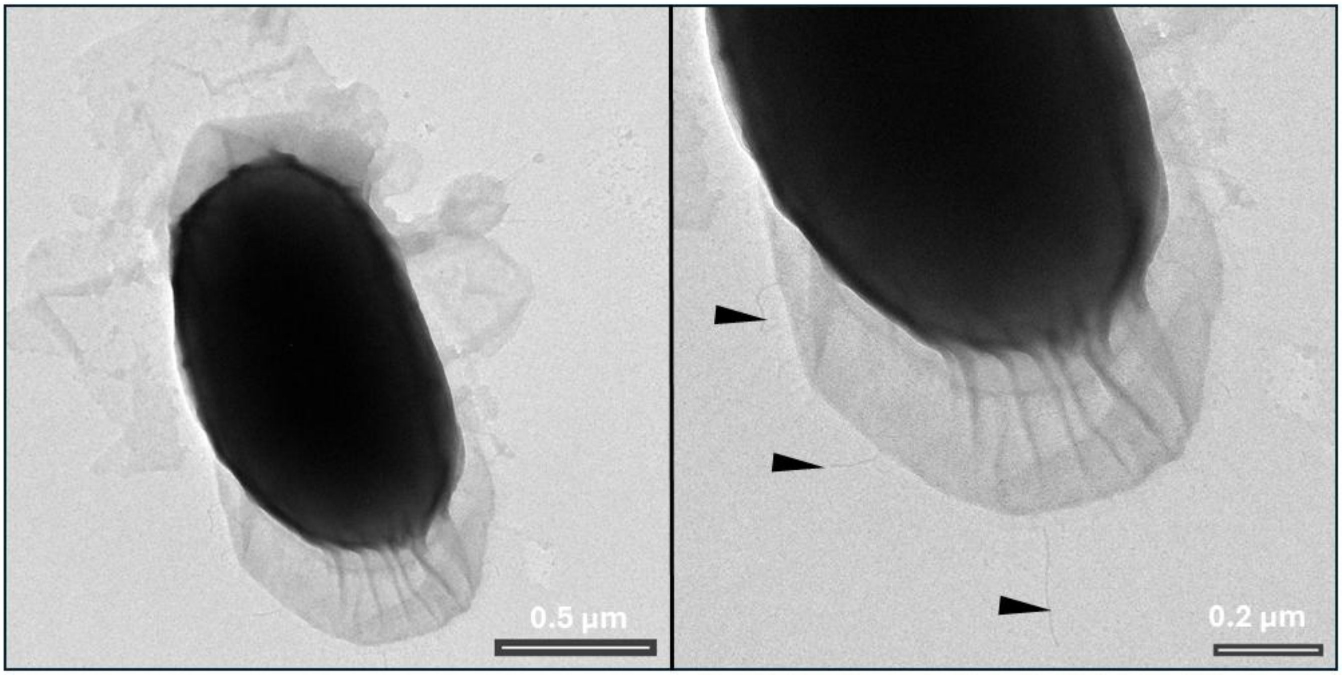

Supplementary Fig. S16. nsTEM of *B. paranthracis* Δ*ena1C::pHT304-P$_{ena1C}$-ena1C* (C142S). Black arrows show L-ENA

References

1. Lindbäck, T. *et al.* CodY, a pleiotropic regulator, influences multicellular behaviour and efficient production of virulence factors in *Bacillus cereus*. *Environ Microbiol* **14**, 2233–46 (2012).

2. Arantes, O. & Lereclus, D. Construction of cloning vectors for *Bacillus thuringiensis*. *Gene* **108**, 115–9 (1991).

3. Pradhan, B. *et al.* Endospore Appendages: a novel pilus superfamily from the endospores of pathogenic Bacilli. *EMBO J* https://doi.org/10.15252/embj.2020106887 (2021) doi:10.15252/embj.2020106887.

4. Baldwin, P. R. & Lyumkis, D. Tools for visualizing and analyzing Fourier space sampling in Cryo-EM. *Prog Biophys Mol Biol* **160**, 53–65 (2021).
