# Supplementary material for: Spatiotemporal expression of endospore appendages and cryo-EM insights into Ena1C-mediated S-ENA anchoring in Bacillus paranthracis
Source: Sci Rep. 2026 Feb 3;16:7122. doi: 10.1038/s41598-026-38321-0 (PMC12921279; doi:10.1038/s41598-026-38321-0)
Supplement: Supplementary file 8 — Supplementary Movie Information. [file 41598_2026_38321_MOESM8_ESM.pdf]

**Supplementary Movie Information**

# Spatiotemporal Expression of Endospore Appendages and Cryo-EM Insights into Ena1C-Mediated S-ENA Anchoring in *Bacillus paranthracis*

Ephrem Debebe Zegeye[1, *], Mike Sleutel[2, 3], Unni Lise Jonsmoen[1], Jingqi Chen[4, †], Luiza P. Morawska[4], Yohannes Beyene Mekonnen[1], Oscar P. Kuipers[4], Han Remaut[2, 3] and Marina Aspholm[1, *]

[1]Department of Paraclinical Sciences, Faculty of Veterinary Medicine, Norwegian University of Life Sciences (NMBU), 1433 Ås, Norway. [2]Structural Biology Brussels, Vrije Universiteit Brussel, Brussels, Belgium. [3]Structural and Molecular Microbiology, Structural Biology Research Center, VIB, Brussels, Belgium. [4]Molecular Genetics Group, Groningen Biomolecular Sciences and Biotechnology Institute, University of Groningen, Groningen, The Netherlands. † Current affiliation: Department of Chemistry and the Carl R. Woese Institute for Genomic Biology, University of Illinois at Urbana-Champaign, Urbana, USA

*Correspondence: marina.aspholm@nmbu.no; ephrem.debebe.zegeye@nmbu.no

**Supplementary Movie Information. Time-lapse imaging of ENA expression.**

Supplementary Movie **1**. Ena1A-sfGFP (*B. paranthracis ena1A-sfGFP*)

Supplementary Movie **2**. Ena1B-sfGFP (*B. paranthracis*::pHT304-$P_{ena1AB}$-*sfGFP-ena1B*)

Supplementary Movie **3**. Ena1C-sfGFP (*B. paranthracis ena1C-sfGFP*)

Supplementary Movie **4**. Ena3A-sfGFP (*B. paranthracis ena3A-mKate*).

Supplementary Movie **5**. Co-expression of Ena1A-sfGFP and mKate-Ena3A (*B. paranthracis ena1A-sfGFP*::pHT304-$P_{ena3A}$-*mKate-ena3A*).

Supplementary Movie **6**. Co-expression of Ena1C-sfGFP and mKate-Ena3A (*B. paranthracis ena1C-sfGFP*::pHT304-$P_{ena3A}$-*mKate-ena3A*).

Images were acquired every 12 min in Supplementary Movie **1** and **4**, and every 10 min in Supplementary Movie **2**, **3,** **5** and **6**.
